# Photoisomerization Paths of α,ω-Diphenylpolyenes: Reaction Rate Dependence on Temperature, Excitation Wavelength, and Deuteration

**DOI:** 10.1021/jacs.4c09134

**Published:** 2024-11-12

**Authors:** Alexander L. Dobryakov, Daria Schriever, Martin Quick, J. Luis Pérez-Lustres, Ilya N. Ioffe, Sergey A. Kovalenko

**Affiliations:** † N. N. Semenov Federal Research Center of Chemical Physics, Russian Academy of Science, Moscow 119991, Russia; ‡ Department of Physics, Free University of Berlin, Berlin 14195, Germany; § Department of Chemistry, Humboldt University of Berlin, Berlin 12489, Germany; ∥ Department of Chemistry, Lomonosov Moscow State University, Moscow 119991, Russia

## Abstract

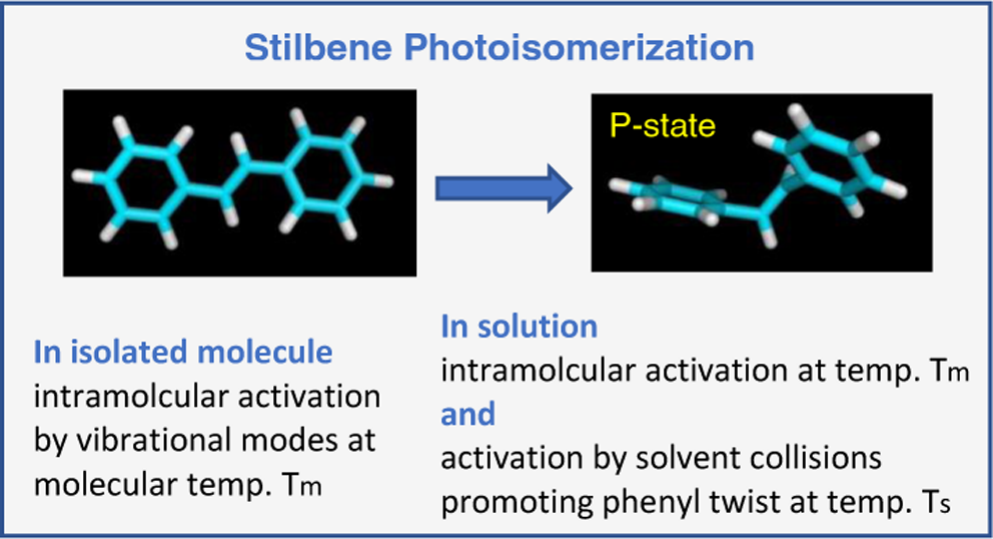

The photoisomerization rate *k*_iso_ of *trans*-stilbene (tS) and trans–trans-diphenylbutadiene (ttD) is studied in solution and compared to that in jet/gas. Rice–Ramsperger–Kassel–Marcus (RRKM) theory correctly predicts the tS rate in jet, *k*_RRKM_ = *A*_m _exp(−*E*_in_/*kT*_m_) with *E*_in_ = 1398 cm^–1^, and *A*_m_ = 1.8 ps^–1^ corresponding to frequency ν_iso_ = 60 cm^–1^ of the reactive mode, *T*_m_ being the molecular temperature. However, the behavior in solution cannot be explained by the RRKM rate alone. In solution the rate *k*_iso_ = *A*_S _exp(−*E*_b_/*kT*_S_) has a similar form, but depends mainly on solvent temperature *T*_S_ and proceeds much faster, *A*_S_ = 19 ps^–1^, *E*_b_ = 1520 cm^–1^ in *n*-hexane. Moreover, excitation at high excess energy, resulting in molecular temperature *T*_m_ = 607 K, affects the rate only slightly, unlike in jet, and contrary to common theoretical models. The experimental results clearly indicate two isomerization paths in solution: via relatively slow intramolecular activation *A*_m_ ∼ 1 ps^–1^, and by much faster solvent activation *A*_S_ = 18 ps^–1^ due to solute–solvent interactions (collisions). The data in *n*-alkanes confirm previously established power dependence *k*_iso_ ∼ η^α^ on viscosity η, with α = 0.30 for tS, and α = 0.35 for ttD. With *E*_η_ being the viscosity barrier, its contribution to *E*_b_ can be isolated, giving the intramolecular barrier *E*_in_ = (*E*_b_ – α*E*_η_), slightly lower than in jet/gas, probably due to the dispersive/induction interactions in solution.

## 1 Introduction

The photoisomerization of *trans*-stilbene (tS)^1−28^ and trans–trans-diphenylbutadiene (ttD)^29−42^ has long been in the focus of research. The probes were carefully investigated in the gas and liquid phase, both experimentally^1−42^ and theoretically.^3,8,19,43−55^ An account on work before 1991 is given in refs^13,17,34^, and some recent results are reviewed in ref (56). It is currently well established that the isomerization proceeds via phenyl torsion around the ethylenic bond. Upon S_0_ → S_1_ optical excitation, the molecule twists over a barrier to perpendicular conformation *P*, and then relaxes through a conical intersection to ground state S_0_, completing the twist with cis or trans isomer.

The isomerization path and the intermediate *P*-state was proposed by Saltiel in 1967,^1^ and later received a solid theoretical justification.^3,48,50,52,53^ It was originally called a “phantom state” because of the difficulty for detection. Indeed, it took 40 years to observe and spectroscopically identify it first in jet^23^ and then in solution.^24^ The problem was related with its very short, ∼0.1 ps lifetime, lack of emission, and with that the *P* absorption band lay in the UV, not easily accessible in earlier studies. With the progress in ultrafast pulses, and especially in broadband transient absorption spectroscopy,^57^ it became possible to cover the UV range, while keeping a sub-0.1 ps resolution.^24^ Later on, a better record of *P* was achieved with 1,1′-stilbene derivatives in which the *P*-state is long-lived, up to 100 ps.^25^

Starting from early studies, a major challenge was to test Rice–Ramsperger–Kassel–Marcus (RRKM)^3,19,58^ and Kramers^59,60^ theory of unimolecular reactions. At rather general conditions,^3,19^ the RRKM theory predicts an isomerization rate *k*_iso_ in a simple Arrhenius form
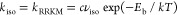
1where ν_iso_ in cm^–1^ is the frequency of the reaction mode, *E*_b_ – isomerization barrier, *k* – Boltzmann constant, *T* – temperature, *c* – velocity of light. To be applicable eq 1 requires fast intramolecular vibrational redistribution (IVR), *k*_IVR_ ≫ *k*_iso_, and low-frequency isomerization mode ν_iso_≪ *kT*. When ν_iso_ > *kT*, the preexponential factor *A* = *c*ν_iso_ is more complex, although still applicable for crude estimates.^3^

Jet experiments on tS at low excess excitation energy reported *k*_iso_ ∼ 1 ns^–1^, *E*_b_ ≈ 1200 cm^–1^.^8−11^ A similar barrier *E*_b_ was also measured in hydrocarbon solution.^2,13,16^ Assuming the reaction frequency ν_iso_ to be independent of the environment, one may expect a comparable photoisomerization rate, however the rate in solution is in fact 1 order of magnitude higher. For example, in *n*-hexane at 293 K, *k*_iso_ > 10 ns^–1^.^13^ Moreover, measurements in buffer gases^14,18^ revealed a linear rise of *k*_iso_ with the buffer pressure, thus directly indicating a crucial role of solute–solvent collisions in promoting the reaction.

There are basically two ways to resolve the dilemma. The first, and currently widely accepted, is that IVR is presumably slow (restricted) at collision less conditions. It limits the energy flow from intramolecular bath to reaction mode, and hence slows down the isomerization rate in jet and low-pressure gases. Once the reaction depopulates the transition state, the slow IVR cannot repopulate it sufficiently fast. By adding a buffer gas, collisions with tS molecules accelerate IVR, and at high-pressure the IVR becomes fast enough to not limit the reaction rate anymore. Early computations reported the reaction frequency ν_iso_ in the range 400–600 cm^–1^,^10,17,43,47,48^ thus supporting the hypothesis of slow IVR. Indeed, with ν_iso_ = 600 cm^–1^ it was possible to fit the reaction rate *k*_iso_ both in solution and in buffer gases at different pressures.^48,51^ It is worth noting that with the barrier *E*_b_ ≈ 1200 cm^–1^,^8−11^ the choice ν_iso_ ∼ 600 cm^–1^ is enforced to match the rate in solution by the RRKM theory.

An alternative solution is to assume that solute–solvent collisions directly activate^14,21,27^ the reactive mode ν_iso_ in addition to intramolecular activation. In 1986 Balk and Fleming^14^ mentioned such a mechanism but abandoned it as inconsistent with the RRKM approach. Instead, they supported the restricted IVR hypothesis.^14,15,43,47,48^

Note that restricted IVR was questioned in 1996 by Zewail and co-workers^11^ who inferred for tS in jet *k*_IVR_ > 1 ps^–1^ ≫ *k*_iso_ at *T* = 293 K. But proponents of the limited IVR^43,47,48,51,54,55^ put forward a much stronger requirement, *k*_IVR_ ≫ *c*ν_iso_, with ν_iso_ = 600 cm^–1^ resulting in *k*_IVR_ ≫ 18 ps^–1^.^47,48,51^ However, Troe and co-workers^19^ demonstrated in 2002 that the complete IVR is consistent with the results in jet.^11^ They successfully fitted the data by the RRKM rate, eq 1 with ν_iso_ = 24 cm^–1^ and *E*_b_ = 1155 cm^–1^. Recent quantum chemical computations^52,53^ likewise support ν_iso_ ∼ 30 cm^–1^. Regarding the rate in solution, Troe and co-workers concluded that “the trans-stilbene enigma of an order of magnitude discrepancy between thermally averaged gas-phase rate and low viscosity liquid-phase rate remains unresolved”^19^ thus leaving the problem for future work.

Kramers^59^ in 1940 considered the viscosity effect on the unimolecular reaction rate. At very low viscosity η the Kramers theory^59,60^ predicts *k*_Kram_ ∼ η, and for higher η the rate is expressed as
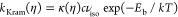
2where coefficient κ(η) is given by
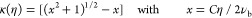
3Here *C* is a constant, and ν_b_ the reaction frequency at the barrier top. When *x* ≪ 1 the viscosity is still small and the RRKM rate *k*_RRKM_ is recovered. For high viscosity, *x* ≫1, κ ≈ 1/2*x* and *k*_Kram_ ∼ *k*_RRKM_/η is inversely proportional to η, that seems reasonable in the liquid phase. There were numerous attempts to fit the photoisomerization rate *k*_iso_ of α,ω-diphenylpolyenes by the Kramers eq 2, but only with a partial success.^6,12,29,33,37,38^

It is worth mentioning an important difference between tS and ttD regarding their electronic level structure. For tS the lowest S_1_ state is always 1^1^B_u_ both in gas and liquid.^49,52,53^ However, this is not the case for ttD, its S_1_ state in gas is 2^1^A_g_^32^ and switches to 1^1^B_u_ in solution.^38−41^ Therefore, a comparison of gas- and solution-phase isomerization rate of ttD is meaningless. Nonetheless, a comparison between the rates of tS and ttD in solution is in order, as the isomerization proceeds from the same 1^1^B_u_ state of similar electronic structure. For higher α,ω-diphenylpolyenes, the dark 2^1^A_g_ becomes the lowest S_1_ state also in solution^34^ complicating spectroscopy of these probes. In this regard the photochemistry of stilbene is unique among α,ω-diphenylpolyenes.

Next important note concerns the rate dependence *k*_iso_ on the excitation wavelength λ_exc_. In jet/gas at collisionless conditions, the dependence on λ_exc_ is very pronounced,^11^ while in solution it is rather weak. This was naturally ascribed to rapid solute–solvent energy transfer,^6,12^ but the consequences for the RRKM and Kramers model were not recognized until 2013.^27^ Briefly, the point is as follows. For a molecule excited high above the 0–0 transition, intramolecular vibrational energy increases resulting in high molecular temperature *T*_m_. In jet/gas at collisionless conditions *T*_m_ remains constant during the isomerization, while in solution the molecule cools down by surrounding solvent molecules to solvent temperature *T*_S_. The cooling dynamics is currently well-known.^20−22^ In particular for tS in aprotic solvents, the cooling time τ_c_ is about 10 ps^22^ that can be easily resolved by modern ultrafast techniques. Consequently, as isomerization and vibrational cooling of tS occur on a comparable time scale, one has to consider the effect of time-dependent molecular temperature *T*_m_(*t*) on the isomerization rate *k*_iso_. That is, one has to measure *k*_iso_(*t*) and compare it to *k*_RRKM_(*t*) or *k*_Kram_(*t*), eqs 1 or 2, under condition of nonstationary *T*_m_(*t*).

An important piece of information can be obtained with deuterated probes. For tS the rate *k*_iso_ was measured and calculated for various deuteration patterns: at the ethylenic bond (D2), phenyl rings (D10), and with full deuteration (D12).^10,15,17,19,43,44,48^ Interestingly, the behavior in jet was qualitatively different from that in buffer gases or in solution. In jet, the rate gradually decreased in the order D0 (nondeuterated), D10, D2, D12, while in solution the rate is the same in D0 and D10, and in D2 and D12, being 1.4 times higher in the former pair.

A theoretical consideration of the photoisomerization path suggests either the commonly assumed twisting motion aka one-bond flip, or various hula-twist/bicycle-pedal mechanisms^23^ which do not involve any significant displacement of bulky phenyl rings, and thus might be possible even in solid matrices. However, there is no evidence that such a motion is realized in stilbene or diphenylbutadiene. Recent computations on cis,cis-diphenylbutadiene in crystalline environment predicts an activation energy of 10,000 cm^–1^,^61^ much higher than the isomerization barrier observed for tS o ttD in solution. Thus, the twisting photoisomerization pathway can be regarded as prevailing, and quantum chemistry helps to understand it in detail, at least in the gas phase.^53^ In solution, however, although the qualitative isomerization picture is likely the same, quantitative simulations of the excited-state evolution are challenging. Indeed, in the *P*-state region of S_1_, a very strong spontaneous polarization develops, and at the same time there is a need to account for both static and dynamic electronic correlation. On the contrary, the Franck–Condon S_1_-state is nonpolar and dominated by a single HOMO–LUMO excitation. Hence, the solvent field should lower the barrier due to high polarity of the *P*-state. Simulations of the deuteration effects on the isomerization rate in excited tS by the transition state theory (TST), thus implying complete IVR,^53^ have been found to agree with the above experimental liquid-phase picture.

All the issues outlined above are addressed in the present paper. Broadband transient absorption spectroscopy is applied to measure the photoisomerization rates *k*_iso_ of tS and ttD in solution at different solvent temperature *T*_S_ and viscosities. In addition, the dependence on excitation wavelengths λ_exc_, that is on molecular temperature *T*_m_, is measured and analyzed. Finally, the rate dependence on the deuteration pattern is also measured and discussed. The results are compared to those in jet/gas.^11^ We show that the RRKM theory works well in jet/gas at collisionless conditions but fails to explain the behavior in solution. Here we suggest a new isomerization mechanism involving solvent collisions with the solute phenyl ring.

## 2 Methods

### 2.1 Quantum Chemical Calculations

The S_1_ and S_2_ states of ttD are computed with the Firefly quantum chemistry package, version 8.2.1,^62^ partly based on the GAMESS(US)^63^ source code. We employ the XMCQDPT2^64^ multiconfiguration quasi-degenerate perturbation theory previously applied by us to tS.^53^ The (12e,12o) active space includes all π and π* orbitals except for two lowest and two highest ones. In order to cover the major contributions to S_1_ and S_2_ as revealed by the PT2 treatment, the state-averaging at the CASSCF level includes seven lowest roots. The XMCQDPT2 model space further encompasses 12 additional roots to ensure convergence of geometry optimizations across the potential energy surfaces. At the PT2 level, the chemical core is frozen, and an intruder state avoidance (ISA) shift of 0.02 au is applied. The Def2-TZVPP basis set is used.

### 2.2 Experiment

The transient absorption setup^57,65^ with applications^22,24−28,41,42^ has been described elsewhere. Transient absorption (TA) spectra of tS and ttD are measured in the spectral range 275–690 nm. Magic angle signal Δ*A* = (Δ*A*_||_ + 2Δ*A*_⊥_)/3 is calculated from parallel Δ*A*_II_ and perpendicular Δ*A*_⊥_ polarization. The temporal instrument response is 0.1 ps broad. Multiple 10–20 pump–probe scans are applied to improve the signal-to-noise ratio. Transient anisotropy ρ is given by
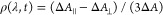
4The anisotropy decay ρ(*t*) is fitted monoexponentially to give the rotational diffusion time τ_R_ (or simply rotational time for brevity).

**Figure 1:**
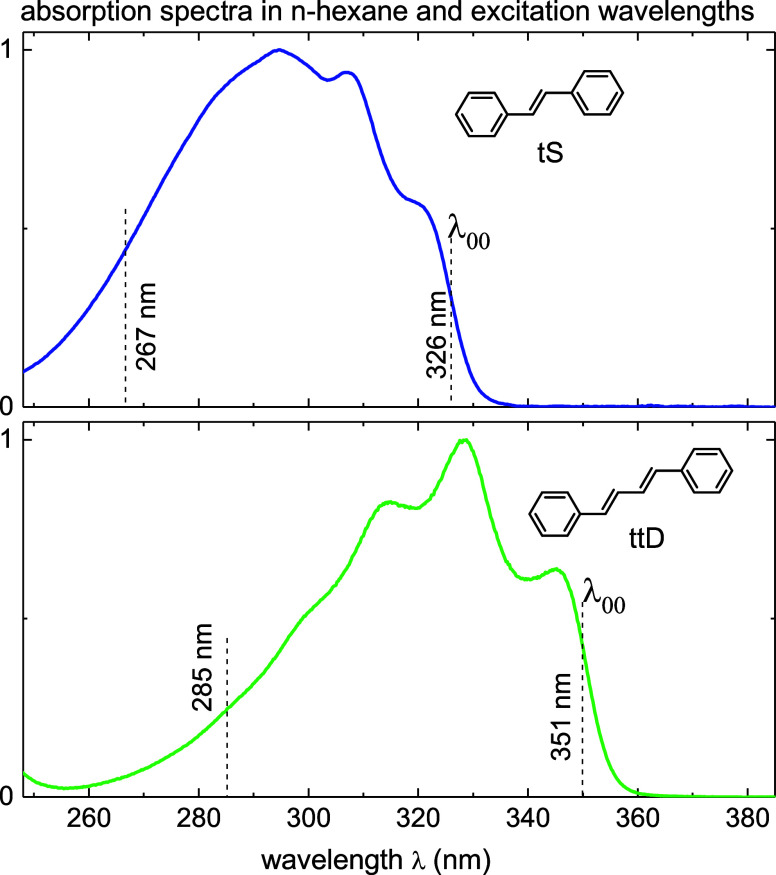
Normalized absorption spectra *A*(λ) of *trans*-stilbene (tS) and trans–trans-diphenylbutadiene (ttD) in *n*-hexane. 0–0 transition λ_00_ and excitation wavelength λ_exc_ for transient absorption (TA) are indicated.

Absorption spectra of tS and ttD in *n*-hexane are shown in Figure 1. In TA measurements, absorbance at λ_exc_ was less than 0.5. A 30 mL solution of 0.1–0.2 mg/mL of tS or ttD was flown through a temperature-stabilized cell of 0.3 mm internal thickness. The pump and probe beams were focused onto the cell at 15° to 0.1 mm spots.

## 3 Results

The plan of this section is as follows. First, we explain how the isomerization rate *k*_iso_ is extracted from broadband TA measurements. Then, viscosity- and temperature-dependent isomerization time τ_iso_ = 1/*k*_iso_ and rotational time τ_R_ is used to obtain the viscosity contribution *E*_η_ to isomerization barrier *E*_b_. This results in intramolecular barrier *E*_in_ in solution that can then be compared to the gas phase barrier. We also consider the isoviscosity rates for obtaining *E*_in_ and conclude that they overestimate the intramolecular barriers. Finally, we present the isomerization kinetics recorded at high excess vibrational energy (λ_exc_ = 267 nm for tS and λ_exc_ = 284 nm for ttD) resulting in hot probe molecules, *T*_m_ ∼ 600 K, in room-temperature solvent, *T*_S_ = 293 K. The results call for a new activation mechanism in the liquid phase.

### 3.1 Transient Absorption Spectra and Photoisomerization Kinetics

**Figure 2:**
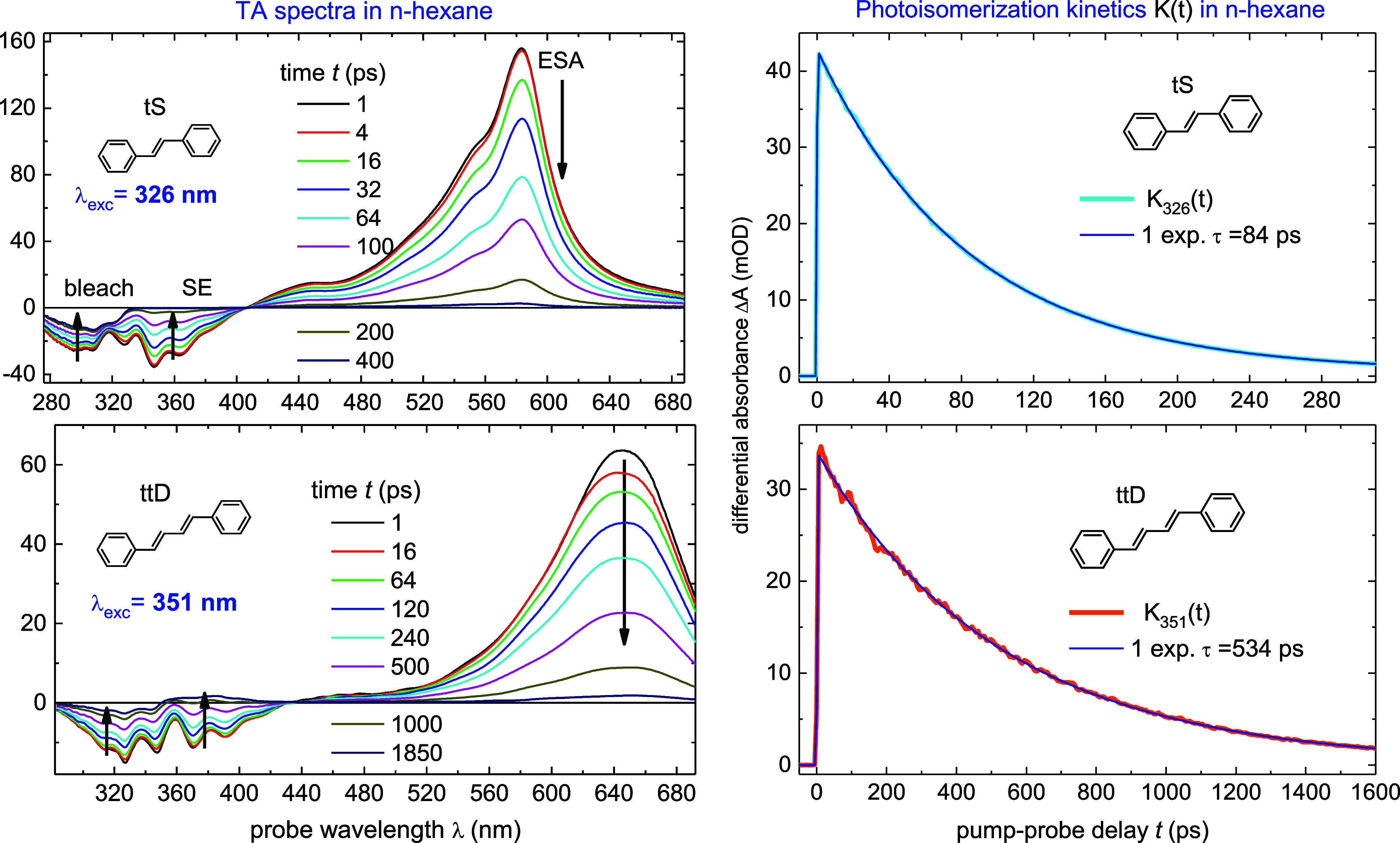
TA spectra upon S_0_ → S_1_ excitation *without* excess vibrational energy in *n*-hexane at *T* = 20 °C, λ_exc_ = 326 nm for tS, and 351 nm for ttD. Bleach and stimulated emission (SE) are negative, excited-state absorption (ESA) is positive. The subns decay of ESA and SE reflect photoisomerization S_1_ → *P* over a barrier to perpendicular state *P*, while subsequent relaxation *P* → S_0_ is barrier less and ultrafast, of ∼0.1 ps. At late time, the cis and trans products are seen in the bleach region. Photoisomerization kinetics *K*(*t*), eq 5, are derived from the ESA decay, fitted monoexponentially with time τ, thus giving the rate *k*_iso_ = 1/τ_iso_ = (1/τ – 1/τ_rad_), with τ_rad_ = 1.6 ns for tS, or 1.5 ns for ttD. See Figures S1 and S2 in Supporting Information for more details.

Typical TA spectra and kinetics of tS and ttD in *n*-hexane, upon excitation *without* excess vibrational energy, are displayed in Figure 2. The excitation wavelength λ_exc_ was centered at the 0–0 transition, 326 nm for tS, and 351 nm for ttD. In that case the molecule preserves its ground-state temperature, and the TA spectra are not disturbed by vibrational cooling in the S_1_ state.^22^ The spectra consist of three well separated bands: bleach, stimulated emission (SE), and excited-state absorption (ESA); bleach and SE are negative, while ESA is positive.

The observed sub-ns decays of ESA and SE mainly correspond to excited-state isomerization S_1_ → *P* to perpendicular conformation *P* over a barrier, while subsequent relaxation *P* → S_0_ is barrier less and ultrafast, of ∼0.1 ps.^24^ Other deactivation paths, like direct (vertical) internal conversion^66,67^ or intersystem crossing, are much slower than the S_1_ → *P* isomerization and can be safely neglected. (For instance, in closely related *trans*-naphtylstilbene the fastest relaxation pathway is radiative, with a lifetime of several ns.^68^) In this picture the bleach recovery develops with the same time constant τ as the decay of ESA. Figures S1 and S2 in Supporting Information confirm that this is indeed the case both for tS and ttD. The bleach signal at late time in Figure 2 corresponds to newly created cis and trans products in S_0_. The photoisomerization dynamics of tS and ttD and of their cis isomers has been studied and analyzed in detail in our previous publications.^24−28,41,53^

The photoisomerization kinetics *K*(*t*) are given by a band integral
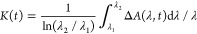
5where the integration is over the ESA region, 450–680 nm for tS, and 540–690 nm for ttD. In case of a monoexponential decay kinetics with time τ, the photoisomerization rate *k*_iso_ is obtained as
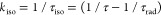
6where τ is the fitted decay time, and τ_rad_ is the radiative time, 1.6 ns for tS,^24^ or 1.5 ns for ttD in solution.^38^

### 3.2 Viscosity Dependence of the Reaction Rate at Room Temperature

**Figure 3:**
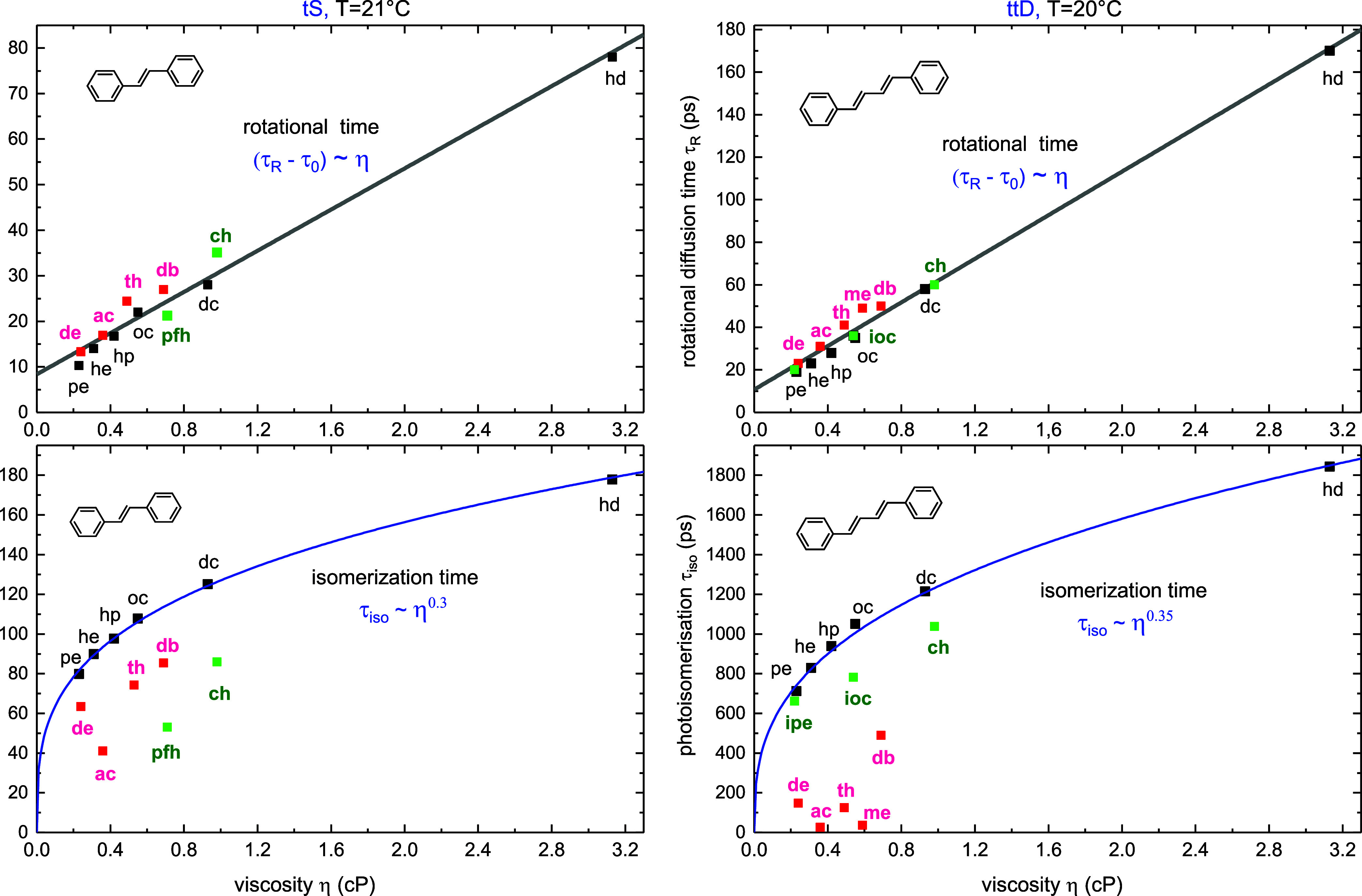
Rotational time τ_R_ (top) and photoisomerization time τ_iso_ (bottom) as a function of solvent viscosity η at 20 °C for tS and 21 °C for ttD. The solvents are listed in Table 1. Note the linear dependence (τ_R_ – τ_0_) ∼ η with τ_0_ = τ_R_(0), and the power dependence τ_iso_ ∼ η^α^ for *n*-alkanes (in black), with α = 0.30 for tS, or α = 0.35 for ttD. Large deviations from the fit for pfh, ch, ioc (in green) possibly indicate molecular size effects, while the deviations in polar solvents (in red) come from lowering the S_1_ → *P* barrier due to polar *P*-state.

We measured isomerization time τ_iso_ and rotational time τ_R_ of tS and ttD for a broad selection of solvents. The results are summarized in Table 1 and depicted in Figure 3. The solvents include *n*-alkanes (black symbols), isoalkanes, (green), perfluorohexane and cyclohexane (also in green), and polar solvents (red).

**Table 1:** Solvent Properties, Rotational Time τ_R_, Photoisomerization Time τ_iso_ at Room Temperature^a^

solvent	ε	*n*	*M* (g/mol)	*D* (g/cm^3^)	*V*_m_ (Å^3^)	*V*_VdW_ (Å^3^)	α (Å^3^)	η (cP)	τ_R_ tS (ps)	τ_iso_ tS (ps)	τ_R_ ttD (ps)	τ_iso_ ttD (ps)
pe C_5_H_12_	1.858	1.357	72.15	0.621	193	96	10.2	0.234	10	80	19	712
ipe C_5_H_12_	1.858	1.357	72.15	0.621	193	96	10.2	0.225			20	661
he C_6_H_14_	1.898	1.375	86.18	0.659	213	113	11.9	0.307	14	89	23	829
hp C_7_H_16_	1.936	1.388	100.2	0.684	245	130	13.8	0.418	17	98	28	938
oc C_8_H_18_	1.965	1.398	114.2	0.699	271	147	15.6	0.547	22	108	35	1051
ioc C_8_H_18_	1.965	1.397	114.2	0.699	271	147	15.6	0.547			36	782
dc C_10_H_22_	2.005	1.412	142.2	0.726	325	215	19.2	0.925	28	125	58	1214
hd C_16_H_34_	2.057	1.435	226.4	0.77	488	283	30.3	3.125	78	178	170	1843
ch C_6_H_12_	2.038	1.426	84.16	0.774	181	102	11.0	0.977	35	83	60	1037
pfh C_6_F_14_	1.582	1.248	338.0	1.691	333	137	12.7	0.708	21	53		
db C_8_H_18_O	3.172	1.399	130.2	0.764	283	153	16.3	0.689	27	85	50	489
de C_4_H_10_O	4.305	1.352	74.12	0.708	174	86	8.98	0.254	13	63	23	147
th C_4_H_8_O	7.729	1.407	72.11	0.884	136	72	7.97	0.53	24	74	41	125
ac C_2_H_3_N	36.69	1.344	41.05	0.776	88	47	4.44	0.357	17	41	31	26
me CH_4_O	33.65	1.328	32.04	0.791	68	36	3.3	0.587			49	36

a 21 °C for tS, 20 °C for ttD. Solvents: *n*-pentane (pe), isopentane (ipe), *n*-hexane (he), *n*-octane (oc), isooctane (ioc), *n*-decane (dc), *n*-hexadecane (hd), cyclohexane (ch), *n*-perfluorohexane (pfh), *n*-dibutylether (db), *n*-diethyl ether (de), tetrahydrofuran (th), acetonitrile (ac), methanol (me). ε, *n* dielectric constant and refractive index, *V*_m_ = *M*/*DN*_A_ solvent volume per molecule, *V*_VdW_ molecular van der Waals volume, α polarizability, *N*_A_ Avogadro number.

The rotational time τ_R_ depends linearly on viscosity η
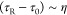
7where τ_0_ = τ_R_(0) is obtained from a linear fit at η = 0. This gives for tS τ_0_ = 8 ps, and for ttD τ_0_ = 10 ps, that is close to free molecular rotation time  = 15 ps at 293 K where *I*_m_ is the moment of inertia of tS or ttD. Note, the fit includes all the solvents measured, that is, τ_R_ depends linearly on viscosity η and is not affected by other solvent properties.

Next, the photoisomerization times τ_iso_ are shown in lower panel of Figure 3. Here a good fit is possible through *n*-alkanes
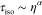
8with α = 0.30 for tS, and α = 0.35 for ttD.

The dependence (8) was reported previously by many authors.^12,13,16,17,29,30,33^ Deviations from the fit in polar solvents are due to the polar *P*-state that lowers the isomerization barrier *E*_b_.^27−29,41^ This results in decreasing τ_iso_ in solvents of higher polarity from dibutylether to acetonitrile, both for tS and for ttD. The stabilization is stronger for ttD, in agreement with its more polar *P* state.^41^ Note also strong deviations from the fit (8) in nonpolar isoalkanes, cyclohexane and perfluorohexane, which are possibly due to molecular size effects. We return to this point in Section 4.4.

### 3.3 Temperature-Dependent Isomerization Rate and Rotational Rate

**Figure 4:**
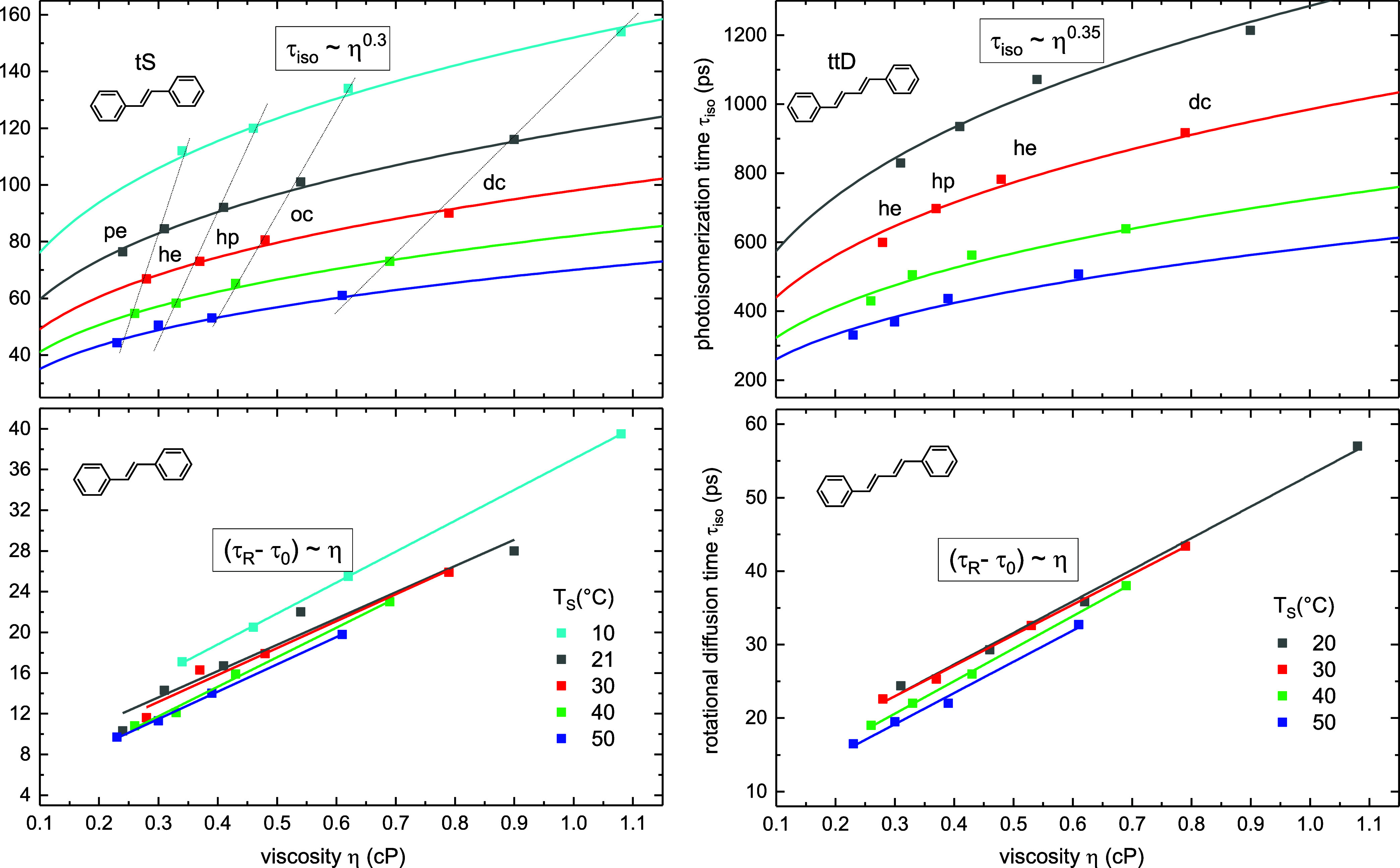
Photoisomerization time τ_iso_ ∼ η^α^ (top) is fitted with α = 0.30 for tS, and α = 0.35 for ttD, for temperatures *T*_S_ = 10, 20, 30, 40, 50 °C. Rotational time τ_R_ (bottom) is fitted linearly (τ_R_ – τ_0_) ∼ η with τ_0_ = τ_R_(0).

We simultaneously measure in the same pump–probe scan the rotational time τ_R_ and the photoisomerization time τ_iso_ at solvent temperature *T*_S_ = 10, 20, 30, 40, 50 °C. The results are collected in Tables 2–5 and displayed in Figure 4. The figure shows that the dependencies (7) and (8) are reproduced with the same fit parameters for all temperatures *T*_S_.

**Table 2:** tS Photisomerization Time τ_iso_ (ps)^a^

*T*_S_ (K)	he	hp	oc	dc	hd	ch	ac	D2 ac
283	120	130	146	170		110	51	70
293	89	97	108	125	178	86	40	55
303	69	76	84	95	130	74	33	44
313	56	60	68	76.5	95	60	28	36
323	45	52	55	63	73	50	23	

a D2 – tS deuterated at the ethylenic bond.

**Table 3:** tS Rotational Time τ_R_ (ps)

*T*_S_ (K)	he	hp	oc	dc	hd	ch	ac	D2 ac
283	17	21	26	40	46	22	21	21
293	14	17	22	28	38	17	18	17
303	12	16	18	26	28	15	15	15
313	11	12	16	23	22	13	14	14
323	10	11	14	20	20	11	11	

**Table 4:** ttD Photoisomerization Time τ_iso_ (ps)

*T*_S_ (K)	he	hp	oc	dc	hd	ch
293	830	935	1071	1214	1843	1034
303	599	697	782	917	1204	715
313	430	507	562	639	832	531
323	331	369	436	507	682	391

**Table 5:** ttD Rotational Time τ_R_ (ps)

*T*_S_ (K)	he	hp	oc	dc	hd	ch
293	24	28	36	57	170	62
303	23	25	33	43	111	49
313	19	22	26	38	81	39
323	17	19	22	33	68	33

**Figure 5:**
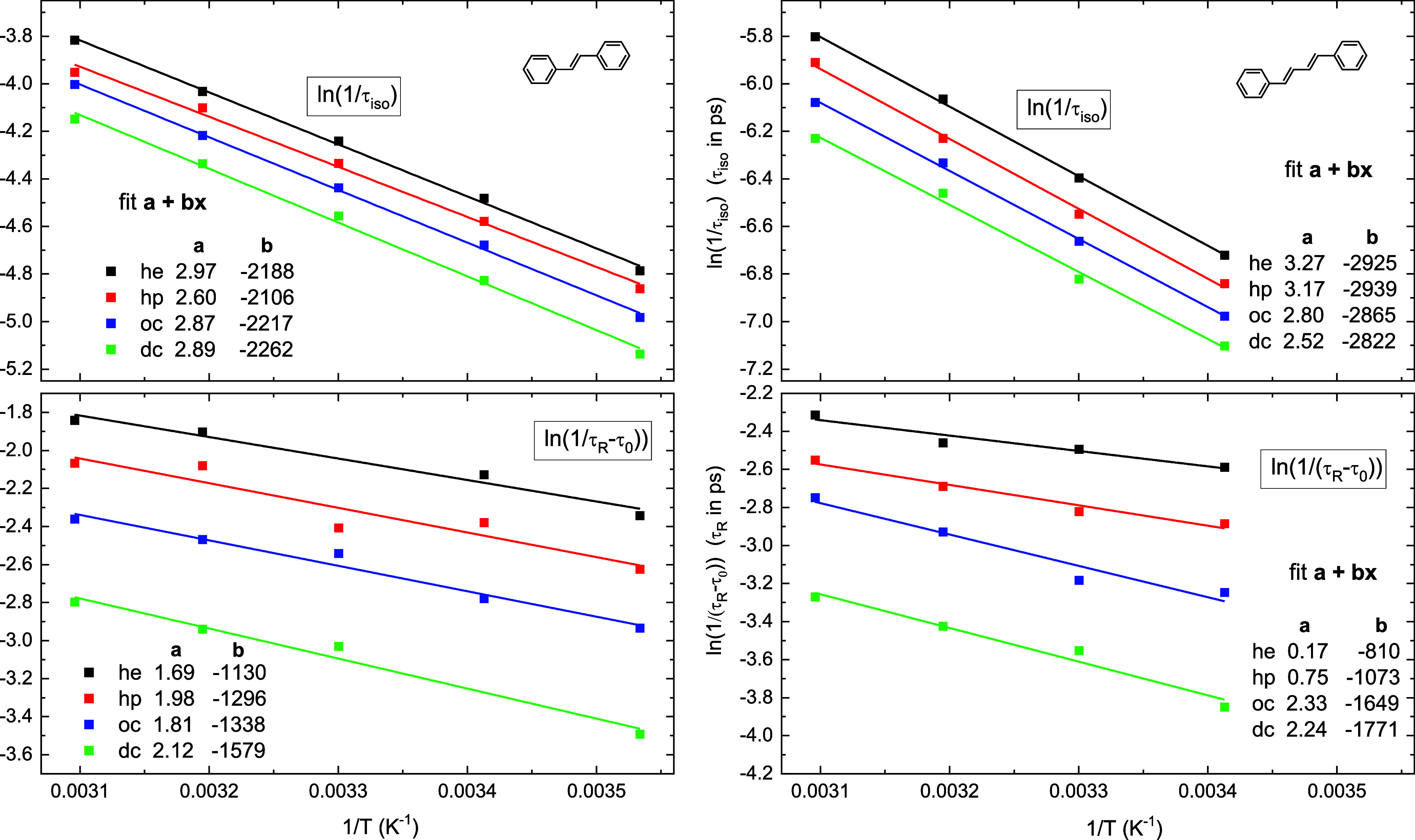
Arrhenius fits of photoisomerization rate *k*_iso_ = 1/τ_iso_ = *A *exp(−*E*_b_/*kT*_S_) and of modified rotational rate *k*_R_ = 1/(τ_R_ – τ_0_) ∼ exp(−*E*_η_/*kT*_S_), *T*_S_ = 10, 20, 30, 40, 50 °C. Slopes **b** give isomerization/rotation barriers *E*_b,η_ = −*R·***b** in kJ/mol, *R* = 8.314 J/K/mol, and intercept **a** gives preexponential factor *A* = e^a^. From τ_iso_ ∼ η^α^ and (τ_R_ – τ_0_) ∼ η one gets the inner barrier *E*_in_ = (*E*_b_ – α*E*_η_) in solution.

Figure 5 shows Arrhenius fits of photoisomerization rate *k*_iso_ = 1/τ_iso_ and of modified rotational rate 1/(τ_R_ – τ_0_). A good linear dependence confirms the Arrhenius behavior of the rates, *k*_iso_ = *A* exp(−*E*_b_/*kT*) and 1/(τ_R_ – τ_0_) ∼ exp(−*E*_η_/*kT*). Note, the barrier *E*_b_ includes both the viscosity contribution α*E*_η_ and the intramolecular barrier *E*_in_. The slope **b**, as indicated in the insets of Figure 5, provides isomerization/viscosity barriers *E*_b,η_ = −*R·***b** (in kJ/mol, *R* = 8.314 J/K/mol), and the intercept **a** gives preexponential factors *A* = e^**a**^. Furthermore, the dependencies τ_iso_ ∼ η^α^, (τ_R_ – τ_0_) ∼ η allow for determining the viscosity contribution α*E*_η_ to barrier *E*_b_, and deriving the inner (intramolecular) barrier in solution
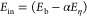
9Although the viscosity contribution α*E*_η_ is obtained for *n*-alkane only, we believe that eq 9 can also be used for polar solvents and branched alkanes, at least as a rough estimate.

The Arrhenius fit parameters are collected in Table 6 for tS, and in Table 7 for ttD. It follows that the intramolecular barrier *E*_in_ closely matches the gas-phase barrier obtained by Zewail and co-workers^8−11^ for tS (Table 6). The barrier difference between the gas phase and alkanes of 160 cm^–1^ may be due to the stabilization of polar *P* by the dispersive and induction interaction in nonpolar solvents.^69^ For ttD, a comparison between the gas and solution reaction rate is not appropriate as the S_1_ state switches from 2^1^A_g_ in jet/gas to 1^1^B_u_ in solution. Remarkably, for tS in *n*-alkanes the preexponential factor *A* is about 10 times larger than in the gas phase. Important consequences from this observation are discussed in Section 3.6.

**Table 6:** tS, Arrhenius Fit Parameters^a^,^b^

	η (cP)	*A* (ps^–1^)	*E*_b_ (cm^–1^)	*E*_η_ (cm^–1^)	*E*_in_ (cm^–1^)
ac	0.36	13.1	1277	920	1001
he	0.307	19.4	1520	904	1249
hp	0.418	13.5	1464	961	1176
oc	0.547	17.6	1541	970	1250
dc	0.925	17.9	1572	1009	1269
mean over alkanes					1236 ± 36
jet/gas^11^		1.8^b^ ± 0.2			1398^b^ ± 26
jet/gas^19^		0.74			1155

a *E*_in_ = (*E*_b_ – 0.30·*E*_η_).

b This work.

**Table 7:** ttD, Arrhenius Fit Parameters^a^

	η (cP)	*A* (ps^–1^)	*E*_b_ (cm^–1^)	*E*_η_ (cm^–1^)	*E*_in_ (cm^–1^)
he	0.307	26	2033	563	1836
hp	0.418	24	2043	746	1782
oc	0.547	16	1991	1146	1590
dc	0.925	12	1961	1231	1530
mean					1680 ± 130
jet/gas^19,32^		0.86			1000

a *E*_in_ = (*E*_b_ – 0.35·*E*_η_).

### 3.4 Isoviscosity Rate

**Figure 6:**
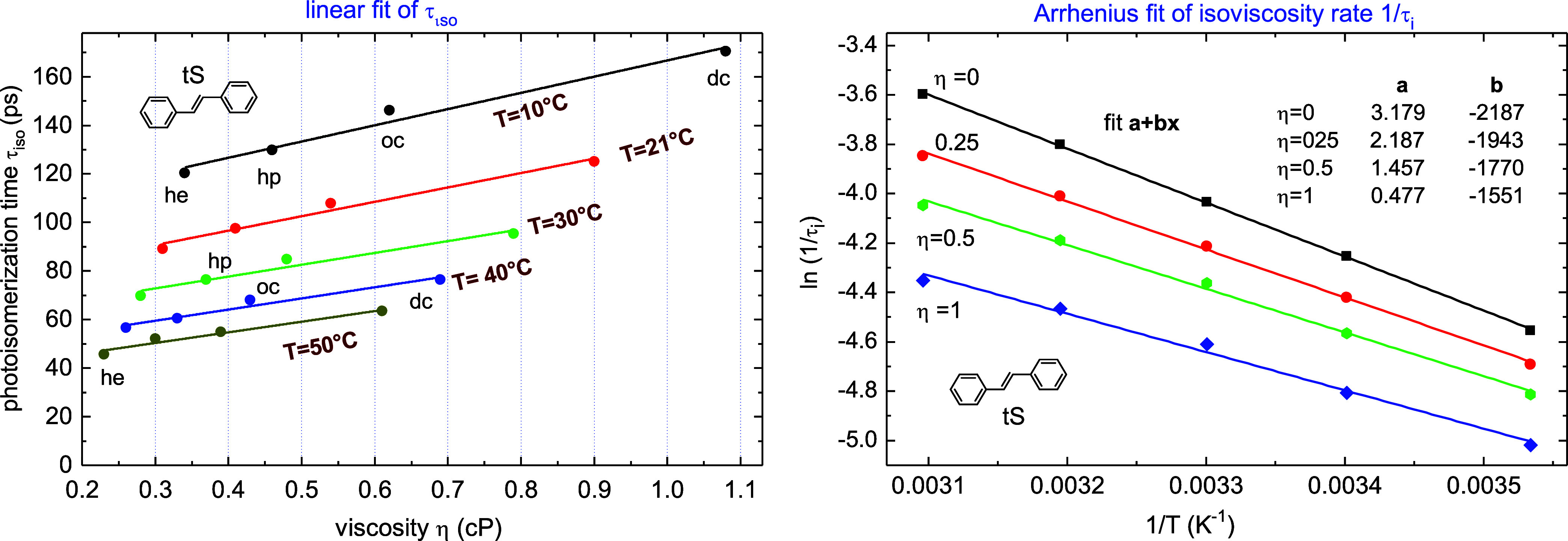
Linear fits of τ_iso_ for *n*-alkanes (left) allow for isoviscosity times τ*_i_* at given viscosity η (right). Arrhenius fits of isoviscosity rates *k_i_* = 1/τ*_i_* = *A*  exp(−*E_i_*/*kT*_S_) result in *higher* barriers *E_i_* for lower η, leading to unphysical results.

We consider now the so-called isoviscosity rate^16,17^ s originally introduced to eliminate the viscosity dependence in *k*_iso_ and get thus the intramolecular barrier *E*_in_. We obtain these rates by fitting τ_iso_(η) in *n*-alkanes in the range 0.2 < η < 1.1 cP as illustrated in Figure 6 (left). We then get isoviscosity times τ*_i_* from the fits at a given viscosity and temperature. Arrhenius fits of such obtained isoviscosity rates (for η = 0, 0.25, 0.5, 1 cP) are shown in Figure 6 (right), where the slope **b** tends to *increase* with decreasing η. In other words, the intramolecular barrier *E*_in_ appears to become higher by lowering η, which seems unphysical. We therefore rely on our estimate of the viscosity contribution α*E*_η_ to *E*_b_ (Section 3.3) in order to derive the intramolecular barrier *E*_in_ in solution.

### 3.5 Excitation Wavelength Dependence in Jet/Gas

**Figure 7:**
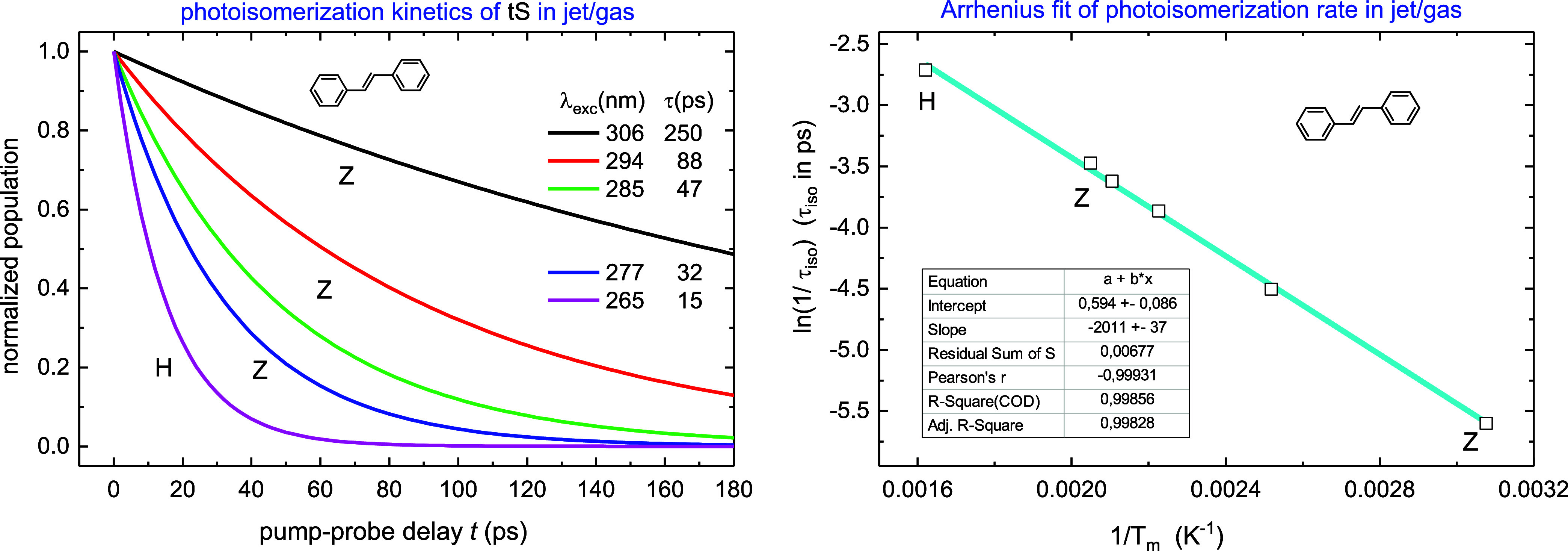
Photoisomerization kinetics of tS in jet/gas (at left) from Zewail (Z, ref (11).) and Hochstrasser (H, ref (5).) with different λ_exc_. The decay time τ strongly depends on λ_exc_ and shortens from 250 ps (306 nm) to 15 ps (265 nm), giving the isomerization rate *k*_iso_ = 1/τ_iso_ = (1/τ – 1/τ_rad_) with τ_rad_ = 3.2 ns. An Arrhenius fit to *k*_RRKM_ = *A*_m_ exp(−*E*_in_/*kT*_m_) results in *E*_in_ = (1398 ± 26) cm^–1^, *A*_m_ = (1.81 ± 0.16) ps^–1^ or ν_iso_ = (60 ± 5) cm^–1^ (at right). Molecular temperature *T*_m_ is calculated by eq 10, *E*_th_ = 1870 cm^–1^ at 294 K (Z), or *E*_th_ = 3497 cm^–1^ at 390 K (H).

Our analysis of the gas-phase photoisomerization is largely based on results by Zewail and co-workers.^11^ They measured the photoisomerization of tS at collisionless conditions with λ_exc_ = 306, 294, 285, 280, 277 nm, and derived both IVR rates *k*_IVR_ and reaction rates *k*_iso_. Their isomerization kinetics, reproduced in Figure 7 at left, reveal a strong dependence on λ_exc_. Here the magenta curve with λ_exc_ = 265 nm is by Hochstrasser and co-workers.^5^ The jet/gas kinetics shall be compared to the kinetics in solution in Section 3.6.

The IVR rate was determined to be faster than 1 ps^–1^.^11^ As the shortest isomerization time is 15 ps, this suggests that the IVR is complete in jet/gas, and molecular temperature *T*_m_ can be calculated from the energy distribution over molecular vibrational modes ν_j_
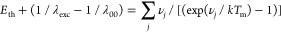
10where *E*_th_ = 1870 cm^–1^ is molecular thermal vibrational energy at 294 K, and λ_00_ = 310 nm is the 0–0 transition for tS in jet/gas. Vibrational frequencies ν*_j_* in S_1_ are obtained by quantum chemical computations (see Supporting Information), and the isomerization rate *k*_iso_ = (1/τ – 1/τ_rad_) is calculated with τ_rad_ = 3.2 ns in gas.^9^

An Arrhenius fit to eq 1 of *k*_iso_ is shown in Figure 7 at right. The linear fit is of good quality and results in ν_iso_ = (60 ± 5) cm^–1^, *E*_b_ = *E*_in_ = (1398 ± 26) cm^–1^, with a preexponential factor *A*_m_ = *c*ν_iso_ = (1.81 ± 0.16) ps^–1^. Thus, the jet/gas data^5,11^ agree with the RRKM eq 1 and deliver the key quantities *E*_in_ and ν_iso_.

### 3.6 Excitation Wavelength Dependence in Solution

**Figure 8:**
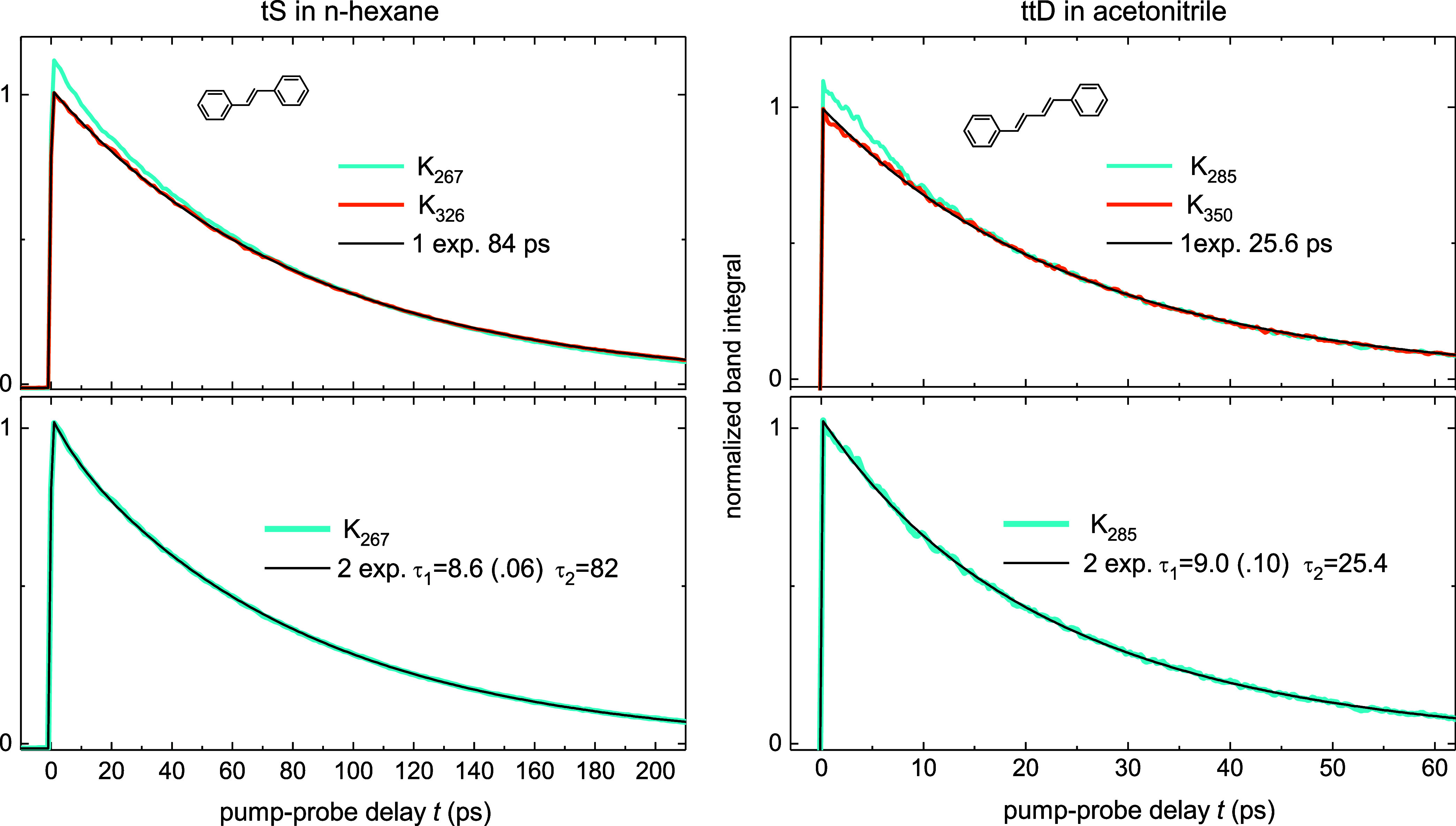
Photoisomerization kinetics *K*_λ_(*t*) where the subscript indicates different λ_exc_. When λ_exc_ = λ_00_ (orange) the molecule has no excess vibrational energy, the decay is monoexponential, τ = 84 ps for tS in *n*-hexane, or τ = 26.3 ps for ttD in acetonitrile. At high excess energy (cyan), λ_exc_ = 267 nm for tS, or 285 nm for ttD, the molecule is initially hot, *T*_m_(0) ∼ 600 K, and then cools down to solvent temperature *T*_S_ = 293 K with time τ_c_ ∼ 10 ps. In that case the decay is biexponential, [*a*_1 _exp(−*t*/τ_1_) + *a*_2_ exp(−*t*/τ_2_)], the fast component *a*_1_ reflects the isomerization activated by hot intramolecular vibrational modes, and the slower component *a*_2_ reflects the isomerization with solvent collisional activation at temperature *T*_S_, eq 13.

We now turn to the photoisomerization in solution, recorded at zero and high excess vibrational energy, Figure 8. The photoisomerization kinetics *K*_λ_(*t*) are calculated with eq 5, the subscript indicates λ_exc_. For tS the ESA range 450–680 nm can still be used in eq 5, as the ESA band is completely within the registration window; its blue shift and narrowing in the course of vibrational cooling does not affect the band integral. For ttD, however, the ESA band extends beyond the registration range, and the spectral shift and band narrowing may change the integral. Therefore, the bleach region 300–350 nm is chosen to calculate *K*_λ_(*t*) for ttD.

As seen from Figure 8, the decay in solution is nearly insensitive to λ_exc_, unlike in jet/gas at collisionless conditions, Figure 7. With zero excess vibrational energy (λ_exc_ = λ_00_) the kinetics is monoexponential, τ = 84 ps for tS in *n*-hexane, and τ = 26.3 ps for ttD in acetonitrile. For high excess energy, λ_exc_ = 267 nm for tS, or λ_exc_ = 285 nm for ttD, the behavior (cyan curve) is quite similar, differing by a small extra-decay at early time. A biexponential fit *K*_λ_(*t*) = [*a*_1_ exp(−*t*/τ_1_)+*a*_2 _exp(−*t*/τ_2_)] gives for tS in *n*-hexane τ_1_ = 10 ps, *a*_1_ = 0.06, τ_2_ = 84 ps, and for ttD in acetonitrile τ_1_ = 10 ps, *a*_1_ = 0.10, τ_2_ = 26 ps.

We have to check now if the kinetics, recorded at high excess vibrational energy, can be reproduced by *k*_RRKM_, eq 1, with nonstationary molecular temperature^20,22^
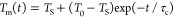
11Here *T*_0_ = 607 K is the initial temperature of tS for λ_exc_ = 267 nm obtained from eq 10, *T*_S_ = 294 K is solvent temperature, and τ_c_ ≈ 10 ps is the molecular cooling time in solution. It was shown^22^ that eq 11 works well in aprotic solvents like *n*-hexane or acetonitrile. The kinetics *K*_λ_(*t*) can then be recast as
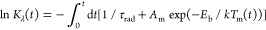
12where *A*_m_ = 19.4 ps^–1^, *E*_b_ = 1520 cm^–1^ should be taken from Table 6.

**Figure 9:**
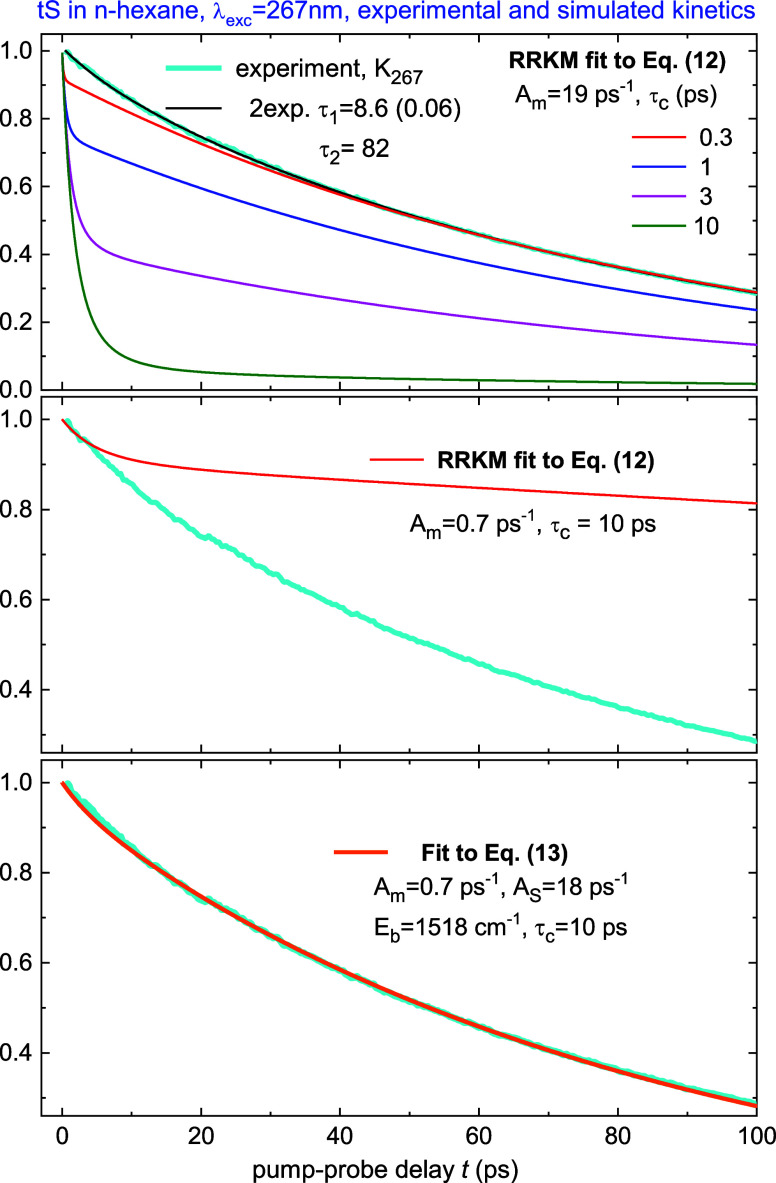
tS photoisomerization kinetics *K*_267_ (cyan) upon excitation at 267 nm in *n*-hexane, its biexponential fit (black), and simulations to RRKM eq 12, or to eq 13. On top panel are the RRKM simulations with *A*_m_ = 19.4 ps^–1^, *E*_b_ = 1520 cm^–1^ (Table 6) and τ_c_ varying from 0.3 to 10 ps, *T*_m_(*t*) is from eq 11 with *T*_0_ = 607 K, *T*_S_ = 294 K. The simulations for τ_c_ > 1 ps strongly deviate from experiment, and even for unreasonably short τ_c_ = 0.3 ps, the early decay cannot be reproduced. The early decay may be reproduced with *A*_m_ = 0.7 ps^–1^ (_m_iddle panel) but then the late behavior deviates considerably. A reasonable fit is possible by eq 13 with *A*_m_ = 0.7 ps^–1^, *A*_S_ = 18 ps^–1^ as shown in low panel.

Figure 9 compares experimental kinetic *K*_267_ for tS to the RRKM simulations by eq 12. Clearly, *k*_RRKM_ cannot fit the experimental curve with any cooling time in the range 0.3 < τ_c_ < 10 ps (top panel). Even for unreasonably short τ_c_ = 0.3 ps, the early decay cannot be reproduced. Alternatively, with *A*_m_ = 0.7 ps^–1^ one may fit the very early behavior but then the late decay substantially deviates (middle panel).

Nonetheless, we have shown in Section 3.5 that eq 1 is correct for isolated tS in jet/gas (Figure 7). Hence, *k*_RRKM_ should also contribute to the rate in solution. Indeed, the photoisomerization kinetics is biexponential, *K*_267_ = [*a*_1 _exp(−*t*/τ_1_) + *a*_2 _exp(−*t*/τ_2_)] where the fast component τ_1_ ≈ τ_c_ reflects the cooling of tS molecule. It is therefore natural to ascribe the fast decay to the intramolecularly activated tS isomerization (molecular contribution). The slower component should then represent the solvent-induced isomerization, as activated by solute–solvent collisions. The interpretation is supported by the results in buffer gases^14,18^ where the rate *k*_iso_ is proportional to the buffer pressure, or to the collision rate of tS and buffer molecules. At high molecular temperature *T*_m_, the intramolecular activation dominates because of a high exponential factor, while at *T*_m_ = *T*_S_ the solvent activation prevails due to a large frequency of solute–solvent collisions. Thus, the photoisomerization rate in solution can be expressed as^27^
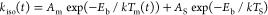
13Here the first term is the intramolecular part, with temperature *T*_m_(*t*) from eq 11. For tS we expect *A*_m_ ∼ 1 ps^–1^ similar as in jet (Figure 7). The second term is the solvent contribution at temperature *T*_S_, induced by solute–solvent collisions, *A*_S_ being the collision rate. Notice, *T*_m_ and *T*_S_ in eq 13 are generally *different* as the probe molecule is heated up upon ultrafast optical excitation. When λ_exc_ = λ_00_ no heating occurs, *T*_m_ = *T*_S_ and eq 13 reads
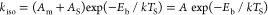
13a

The rate has the same form as eq 1 with *A* = (*A*_m_ + *A*_S_). Taking *A* = 19.4 ps^–1^ in *n*-hexane and *A*_m_ = 1.8 ps^–1^ from Table 6 one gets *A*_S_ = 17.6 ps^–1^. Thus, at room temperature the solvent contribution to the photoisomerization rate is 10 times higher than the intramolecular contribution.

A reasonable fit to eq 13 is obtained with *A*_m_ = 0.7 ps^–1^, *A*_S_ = 18 ps^–1^, *E*_b_ = 1520 cm^–1^, τ_c_ = 10 ps, *T*_0_ = 607 K, *T*_S_ = 294 K, as shown in low panel of Figure 9. The fit is good, indicating the model captures main features of the photoisomerization in solution. Note that the fitted value *A*_m_ = 0.7 ps^–1^ in *n*-hexane corresponds to the reactive mode ν_iso_ = 23 cm^–1^ that is substantially smaller than ν_iso_ = 60 cm^–1^ measured in jet (Figure 7), indicating that the effective isomerization path/frequency in solution is different from that in the isolated molecule.

To sum up this section, eq 13 suggests a new expression for photoisomerization rate in solution, where the reaction is concomitant to vibrational cooling. In jet/gas at collisionless conditions, the molecule is at constant temperature *T*_m_ and isomerizes with constant rate *k*_RRKM_ of eq 1, determined by excess energy (1/λ_exc_ – 1/λ_00_). In solution, *T*_m_(*t*) decreases to solvent temperature *T*_S_, eq 11, the intramolecularly activated isomerization slows down to reveal a new isomerization mechanism, due to activation by solute–solvent collisions. The latter is 10 times more efficient than the intramolecular activation. A possible mechanism behind the collisional activation is that the phenyl rings of tS or ttD upon colliding with solvent molecules acquire a momentum perpendicular to the molecular plane that promotes isomerization about the ethylenic bond.

### 3.7 Deuteration Effect on the Isomerization Rate

**Figure 10:**
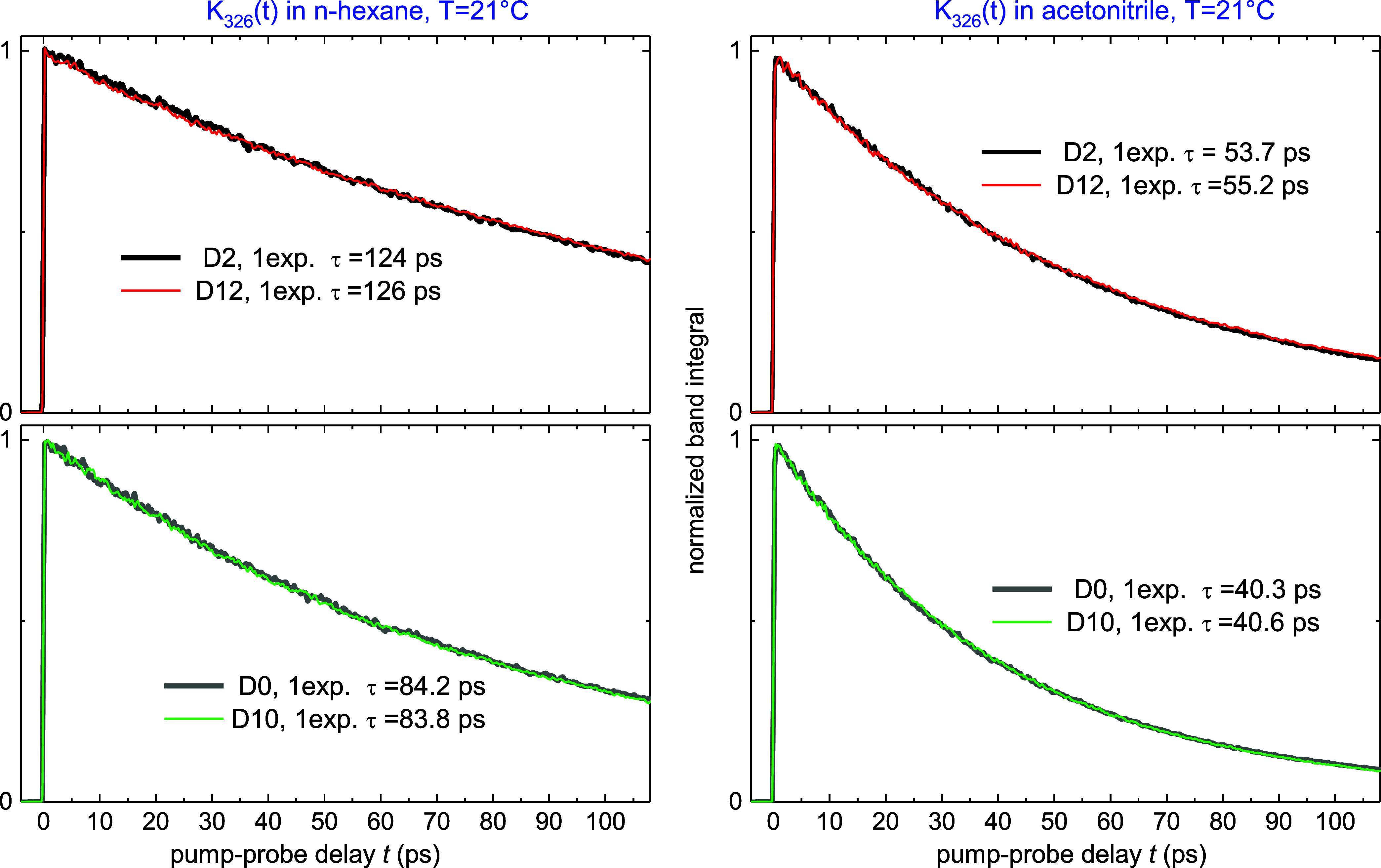
Deuteration effect on the photoisomerization kinetics *K*_326_(*t*) of tS in solution upon λ_exc_= 326 nm. Here D0 nondeuterated tS, D2 ethylenic deuteration, D10 phenyl deuteration, D12 full deuteration. The decays are monoexponential with time constant τ shown as insert. The kinetics are indistinguishable for D0, D10, and for D2, D12, indicating the same *A*_S_ in eq 13a as expected for the collisional isomerization mechanism. The D2 kinetics is slower than the D0 kinetics by factor 1.49 in *n*-hexane and 1.35 in acetonitrile, the effect being due to the different isomerization barrier *E*_b_ (see Figure 11).

Further interesting and corroborating results are obtained with deuterated stilbenes D2, D10, D12 (with ethylenic, phenyl, and full deuteration, respectively, while D0 means no deuteration). Figure 10 displays photoisomerization kinetics *K*_326_ in *n*-hexane and acetonitrile, λ_exc_ = λ_00_ = 326 nm. It is seen that the kinetics is indistinguishable for D0, D10, and for D2, D12. Next, the D2 rate is 1.4-fold slower than that of D0. How can this be explained in view of eq 13?

The solvent contribution *A*_S_ to the rate *k*_iso_ is proportional to the frequency of solute–solvent collisions that depends on the area of phenyl rings and solvent properties. The effect of increased mass on the momentum of the phenyl rings is rather modest even in D12, while in D2 the rings are not affected at all. Therefore, we expect *A*_S_ to be roughly the same for all the isotopomers. Hence, the rate difference of D0 and D2 (and D10, D12) should originate from the difference in barrier *E*_b_.

**Figure 11:**
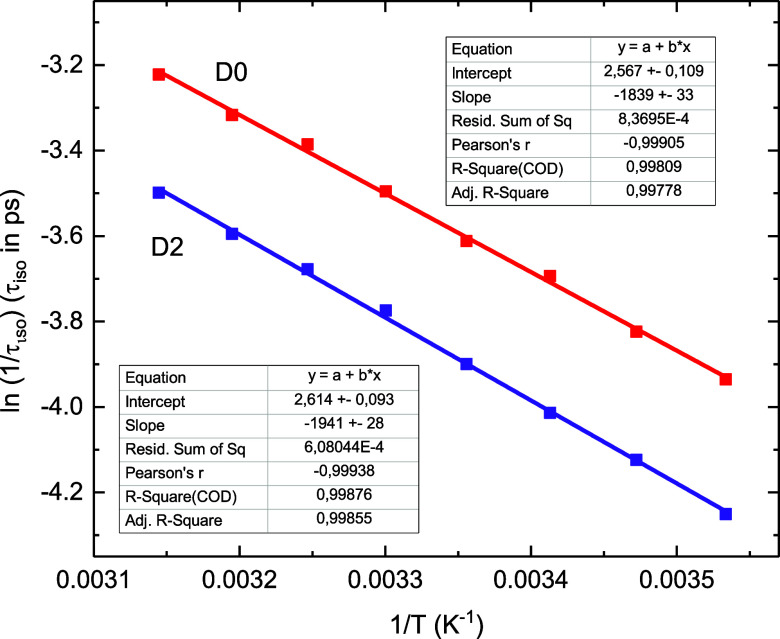
An Arrhenius fit of photoisomerization rate *k*_iso_ = *A*  exp(−*E*_b_/*kT*_S_), *A* = (*A*_m_+*A*_S_), eq 13a, for nondeuterated D0 and deuterated D2 in acetonitrile. The fit results in *A* = (13.0 ± 1.4) ps^–1^, *E*_b_ = (1278 ± 23) cm^–1^ for D0, and *A* = (13.6 ± 1.3) ps^–1^, *E*_b_ = (1349 ± 19) cm^–1^ for D2, that confirms the same collision frequency *A*_S_ for D0 and D2, and the different isomerization barrier *E*_b_.

We have checked this prediction by measuring temperature-dependent rate *k*_iso_ for D0 and D2 in acetonitrile at strictly identical conditions. The result is presented in Figure 11, *A* = (13.0 ± 1.4) ps^–1^, *E*_b_ = (1278 ± 23) cm^–1^ for D0, and *A* = (13.6 ± 1.3) ps^–1^, *E*_b_ = (1349 ± 19) cm^–1^ for D2. This confirms the equality in preexponential factor *A*, and the difference in barrier *E*_b_ due to the isotope effect on the zero-point energy correction, in agreement with our earlier estimates.^53^ The said barrier difference of 71 cm^–1^ in acetonitrile agrees well with that of 67 cm^–1^ reported by Saltiel^70^ for D0, D2 in *n*-hexane.

### 3.8 Two Excited States in ttD

In the gas-phase, the S_1_ state of ttD is 2^1^A_g_ with a significant contribution of the double HOMO–LUMO excitation while the S_2_ state is essentially singly HOMO–LUMO excited 1^1^B_u_.^39,40^ Despite the loss of symmetry upon twisting, we will, for brevity, extend those designations over twisted geometries when referring to the states characterized by the respective electronic contributions. The available experimental gas-phase estimate of the isomerization barrier from 2^1^A_g_ is ca. 1050 cm^–1^,^32^ roughly coinciding with the gas-phase separation between 2^1^A_g_ and 1^1^B_u_.^40^ But in solution the two states reorder, and already in hexane ^1^B_u_ is found about 1200 cm^–1^ below ^1^A_g_.^38,41^ In *n*-hexane, the apparent isomerization barrier increases to *E*_b_ = 1750–2150 cm^–1^,^29,41^ somewhat below the sum of the gas-phase barrier from 2^1^A_g_ and the above solution-phase separation between 2^1^A_g_ and ^1^B_u_. Hence two alternative possibilities can be considered: either the solution-phase isomerization barrier is fully due to 1^1^B_u_, or the solvent field makes 2^1^A_g_ barrierless, and the barrier may then be determined by an interplay of the two states. Since the both states are nonpolar in the Franck–Condon region,^41^ the solvent field may cause a decrease in the barrier height, similarly to the case of tS. Indeed, the experiment reveals much faster photoisomerization kinetics for ttD in polar solvents.^41^

Previously, the experimental barrier *E*_b_ for ttD was fairly well reproduced at the linear response TDDFT level (where 2^1^A_g_ is effectively missing, and 1^1^B_u_ thus remains uncontested as S_1_).^41^ The present gas-phase XMCQDPT2 results on 1^1^B_u_ as S_2_ agree well with those TDDFT findings. The barrier is observed at a twisting angle of 108° (vs 120° at the TDDFT level) where 1^1^B_u_ closely approaches 2^1^A_g_ and they start to interact. In the barrier region, 1^1^B_u_ is only slightly polar, with a dipole moment of 1.9 D. The calculated barrier *E*_b_ = 2650 cm^–1^ with respect to the origin of 1^1^B_u_ is slightly higher than the TDDFT value, but as a gas-phase estimate, not accounting for the *P*-state stabilization and the barrier lowering in solution, it is still consistent with experiment.^41^ Note that the gas-phase origin of 1^1^B_u_ can be established only with symmetry restrictions due to vibronic coupling with 2^1^A_g_.^32^ Previously, we have observed a true, though very shallow, minimum in 1^1^B_u_ at the same XMCQDPT2 level of theory, but such fine details turn out to be highly susceptible to the choice of the model space size.^41^

In 2^1^A_g_ we observe the isomerization barrier at a twisting angle of ca. 91°. Here, the twisting is accompanied by gradually increasing prebarrier polarization associated with pyramidalization of the carbon site next to the phenyl ring. At the transition state, the dipole moment already reaches 7.6 D. Thus, 2^1^A_g_ can, indeed, become barrierless in solution, and may be hence involved in determining the excited-state isomerization barrier *E*_b_. The XMCQDPT2 gas-phase isomerization barrier in 2^1^A_g_ of 2400 cm^–1^ is twice higher than the experimental value.^32^ This discrepancy may reflect the limitations of the available computational accuracy, rather than the existence of some alternative isomerization pathway so far unaccounted for.

## 4 Discussion

### 4.1 RRKM is Correct in Jet/Gas at Collisionless Conditions

We start with the photoisomerization of tS in jet/gas, as shown in Figure 7. A key result here is that the RRKM rate *k*_RRKM_ is consistent with the experimental rate *k*_iso_ = *A*_m _exp(−*E*_b_/*kT*_m_) at collisionless conditions, where molecular temperature *T*_m_ is calculated from λ_exc_ by eq 10. Since eq 10 is valid at the condition of unrestricted IVR, the observed Arrhenius behavior of *k*_iso_ implies that IVR is *complete* in jet/gas.^11^ This agrees with direct measurements of *k*_IVR_ > 1 ps^–1^ by Zewail and co-workers,^11^ and with gas-phase results by Troe and co-workers.^19^ The Arrhenius fit to eq 1 is good and results in reaction frequency ν_iso_ = (60 ± 5) cm^–1^ and barrier *E*_in_ = (1398 ± 26) cm^–1^.

Note that the reactive mode ν_iso_ in jet/gas was not experimentally determined in the past, but was usually derived from computations.^8,10,17,19,44,47,48^ This is because the thermal rate *k*_iso_(*T*) was never applied in previous gas-phase studies. Instead, the workers usually measured and analyzed the microcanonical rate *k*_iso_(E)^8−15,19^ as function of excess energy *E* = (1/λ_exc_ – 1/λ_00_)
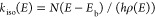
14Here *N*(*E* – *E*_b_) is the number of vibrational states below energy *E* in the transition state (except the reactive mode ν_iso_), ρ(*E*) is the excited-state density of the reactant. It is well-known that the thermal rate *k*_iso_(*T*) can be obtained from *k*_iso_(*E*) by averaging over the thermal distribution^8,19^
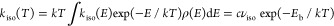
15The second equality is just *k*_RRKM_, eq 1, obtained at condition that the vibrational modes in the transition state are the same as in the reactant, and ν_iso_ ≪ *kT*. The microcanonical rate *k*_iso_(*E*) is less transparent than *k*_iso_(*T*), furthermore *k*_iso_(*E*) can be evaluated only numerically that in practice requires precalculated ν_iso_. However, just this rate was commonly applied in the most of gas-phase works.^9−15,17−19^ Even Troe and co-workers,^19^ who formally considered eq 15, did not derive ν_iso_ and *E*_in_ from the thermal rate *k*_iso_(*T*). A reason for this was probably historical, as in early stilbene studies there were many uncertain molecular parameters, so the workers preferred to deal with originally measured microcanonical rates *k*_iso_(*E*).

Our analysis of the tS data in jet^11^ results in thermal rates *k*_iso_(*T*) that correspond to ν_iso_ = 60 cm^–1^ and *E*_b_ = *E*_in_ = 1398 cm^–1^. These are different from ν_iso_ = 25 cm^–1^, *E*_in_ = 1155 cm^–1^ by Troe and co-workers^19^ because their ν_iso_ was taken by hand from the tS vibrational spectrum, while *E*_in_ was adjusted to fit the experimental rate. In real systems, the approximation of transition state vibrational modes with the reactant modes may be too crude, especially when high-amplitude low-frequency modes are concerned. Thus, the low-frequency twisting modes and the phenyl rotation modes in tS are considerably anharmonic and can actually be coupled, so that ν_iso_ = *A*_m_/*c* in eq 13 should be viewed as only an effective parameter.

Also, our intramolecular barrier in nonpolar solution *E*_in_ = 1236 cm^–1^ is a bit lower than 1398 cm^–1^ in jet, that is probably due to the inductive and dispersive stabilization of the polar *P*-state.^69^

### 4.2 Two Activation Mechanisms of Isomerization in Solution

The photoisomerization rate dependence on λ_exc_ is weak for tS in solution (Figure 8), in contrast to jet/gas (Figure 7). The main result here is that the behavior at high excess vibrational energy cannot be rationalized in frame of RRKM or Kramers theory, eqs 1 and 2. When one assumes, for example, the validity of eq 1, it is straightforward to simulate photoisomerization kinetics *K*_267_(*t*) at λ_exc_ = 267 nm (see eq 12), with molecular temperature *T*_m_(*t*) = *T*_S_ + (*T*_0_ – *T*_S_)exp(−*t*/τ_c_).^22^ As seen from Figure 9, the experimental kinetics cannot be reproduced for any conceivable molecular cooling time τ_c_ in the range 0.3–10 ps.

However, the RRKM rate is correct in jet/gas for the isolated molecule. Therefore, the rate in solution should contain a molecular part. We suggest that this rate is given by the sum of molecular and solvent contribution, eq 13. This model fits well the experimental kinetics *K*_267_ (Figure 9), reconciles the gas- and solution-phase measurements,^11,18^ and further agrees with the results on ttD and deuterated tS (Figures 8, 10, and 11). Note that the collision frequency *A*_S_ does not depend on deuteration and should be the same for all the tS isotopomers. This results in identical isomerization kinetics for D0, D10, and for D2, D12 (Figure 10). Moreover, *A*_S_ is also of the same value for D0 and D2, while the observed difference in the rate *k*_iso_ originates from the barrier mismatch of 70 cm^–1^ (Figure 11). The derived barrier mismatch in D0, D2 agrees with a previous estimate by Saltiel et al.^70^

Next, the collision frequency *A*_S_ is also identical for ttD and tS by similar arguments. Comparison of Table 6 for tS with Table 7 for ttD shows that the agreement is satisfactory at least in *n*-hexane, *n*-octane, and *n*-decane. Lastly, eq 13 is fully consistent with the linear pressure dependence of *k*_iso_ in buffer gases.^14,18^

An important observation for ttD is that its lowest S_1_ state switches from dark 2^1^A_g_ in jet/gas to bright 1^1^B_u_ in solution.^39−41^ That is the photoisomerization occurs in different electronic states, with generally different barrier *E*_in_ and hence different rate *k*_iso_. Thus, for isolated ttD Troe and co-workers^19^ obtained the following estimate, *k*_iso_(*T*) = 6.2 ns^–1^ at 20 °C with barrier *E*_b_ = 1050 cm^–1^.^19,32^ While the experimental rate in *n*-hexane is *k*_iso_ = 1.2 ns^–1^ (Table 7). Thus, the reaction is *slower* in solution, because the 1^1^B_u_ barrier *E*_b_ = 1836 cm^–1^, is substantially higher than that in jet/gas.

We believe that a mechanism behind the collisional activation is that solvent collisions with tS or ttD phenyl rings deliver a momentum perpendicular to the molecular plane, thus promoting the ethylenic twist. Another collisional isomerization mechanism was proposed by Hamaguchi and co-worker^21^ in frame of their dynamic polarization model. They assumed that solvent collisions with the tS ethylenic bond may directly bring the molecule to the polar *P*-state, and in this way induce the isomerization. This mechanism would be difficult to distinguish from the considered here, as the consequences for the photoisomerization rate are similar.

### 4.3 Different Isomerization Path/Frequency in Solution Compared to Jet/Gas

A fit of photoisomerization kinetics in solution by eq 13 at high excess energy (λ_exc_ = 267 nm for tS, or 285 nm for ttD, Figure 8) allows one to derive the intramolecular contribution *A*_m_ not directly accessible in normal measurements with low excess energy, λ_exc_ ≈ λ_00_. The fit results are summarized in Table 8.

**Table 8:** Solvent *A*_S_ and Molecular *A*_m_ Contribution to *k*_iso_ for D0, D2 and ttD

	τ_c_ (ps)	*A*_S_ (ps^–1^)	*A*_m_ (ps^–1^)	ν_iso_ (cm^–1^)	*E*_b_ (cm^–1^)
D0 he	10	18.0	0.7	23	1518
D0 ac	10	12.9	0.2	7	1280
D2 he	10	17.7	0.3	10	1570
D2 ac	10	13.0	0.1	4	1350
ttD ac	10	13.0	0.1	4	1180

The data in Table 8 reveal the barrier lowering in polar solvent due to the *P*-state stabilization and the kinetic isotope effect of deuteration, the latter being reproducible in the calculations.^53^ However, the both effects affect not only *E*_b_ but also *A*_m_, that leaves a number of questions.

First, we see a 3-fold drop of *A*_m_ in acetonitrile compared to *n*-hexane. If one assumes that *A*_m_ = *c*ν_iso_ as in eq 1, it can hardly be solvent-dependent since excited tS remains nonpolar before reaching the barrier.^53^ Second, there is a 2-fold drop in *A*_m_ upon D2 deuteration. One can hardly see, how the ethylenic deuteration would cause such a drop in the twisting frequency, while the effective mass for the twisting motion is mostly due to the phenyl rings. Furthermore, the estimated ν_iso_ in Table 8 becomes exceedingly low in most cases. In view of that, one may suppose that a simplified picture, with fixed ν_iso_ and remaining normal modes unchanged, is not fully relevant. Perhaps, more exact equations of the transition-state theory need to be invoked, where *A*_m_ includes the *kT*/*h* factor and the ratio of partition sums of transition state and reactant. Unfortunately, rationalization of the effect of solvent polarity on *A*_m_ would still remain problematic. The data in Table 8 may imply that the solvent polarity affects some low-frequency modes in the transition state close to the polar *P*-state, but presently there is no obvious model to explain that.

Consistently with our model, the rate *A*_S_ is virtually independent of deuteration, but there is some 1.4-fold drop in *A*_S_ between *n*-hexane and acetonitrile, implying that the collisions in acetonitrile activate twisting less efficiently compared to *n*-hexane. In the gas-phase collision theory, the reaction rate is inversely proportional to the square root of the reduced mass of colliding particles. Taking *m*_Ph_ = 77, *m*_he_ = 86, *m*_ac_ = 41, one obtains a factor of 1.2 in favor of *n*-hexane. Thus, the trend in the solvent rate *A*_S_ is at least in a qualitative agreement with the proposed model.

### 4.4 On Restricted IVR and Cooling by Excitation

Restricted IVR has been considered so far as a solution of stilbene photoisomerization problem.^8,14,15,43,47,48,54,55^ We briefly discuss this view following a paper by Leitner et al.^48^ Their main assumptions are (i) the RRKM rate *k*_RRKM_ is not achieved in jet because of slow (restricted) IVR; (ii) *k*_RRKM_ is realized in liquid solution and in high-pressure buffers; (iii) a high frequency reaction mode, ν_iso_ = 607 cm^–1^ for D0, and ν_iso_ = 475 cm^–1^ for D2, is proposed in order to explain the high isomerization rate in solution, and the difference in rate for D0 and D2.

Point (i) contradicts to the Arrhenius dependence of *k*_iso_ in jet (Figure 7) that shows (a) that *k*_RRKM_ is consistent with experiment, and (b) suggests that IVR is complete. A strong argument against incomplete IVR was provided by Troe and co-workers^19^ with their RRKM calculation over 5 orders of magnitude of the reaction rate in the gas-phase for D2, D10 and D0 (Figures 4 and 5, ref (19)). Point (ii) is refuted by the isomerization kinetics in solution at high excess excitation energy (Figure 9); at *T*_m_(0) = 607 K the early isomerization rate is similar to the gas-phase rate, but then the low temperature behavior cannot be not reproduced. This implies that the reaction is triggered by a process which is faster than IVR and directly activates the reaction mode (like solvent collisions with the phenyl rings in our model). Next, the assumed high frequency ν_iso_ = 607 cm^–1^ (iii) disagrees with the experiment, ν_iso_ = 60 cm^–1^ for D0 in jet. Furthermore, the assumed different ν_iso_ for D0 and D2 would result in the different rate *A* in solution, contrary to the same *A* = 13 ps^–1^ for D0, D2 in acetonitrile, Figure 11.

An alternative explanation for a slow isomerization rate in jet, compared to solution, was proposed by Pollak and co-workers^45,46^ who assumed substantial cooling of the tS molecule upon the 0–0 excitation. Such a cooled molecule would be then heated up by surrounding solvent on a 10 ps scale. The heating should be clearly visible in both TA spectra and kinetics in Figure 2. The ESA band would broaden and shift to the red with increasing temperature *T*_m_(*t*), and the kinetics would be nonexponential, with a rising component at early time. As no such effects are observed, we conclude that the temperature change by optical cooling is negligible upon the 0–0 excitation.

### 4.5 Viscosity Effects

The viscosity dependence of the rate *k*_iso_ was extensively studied^6,7,12,13,16,29,33,37,38^ mainly in connection with *k*_Kram_, eq 2, in hope to fit experimental kinetics by the Kramers model.^59,60^ However, *k*_Kram_ is closely related to *k*_RRKM_ corrected for viscosity η. These rates are identical for small η, and proportional to each other for large η, *k*_Kram_ ∼ *k*_RRKM_/η. Thus, their applicability range should be the same. In particular eq 2 is correct for low-pressure gases and small η, when the collisional contribution to *k*_iso_ is negligible. In high-pressure buffers and in liquid solution, eq 2 cannot be applied alone, and should be replaced by eq 13 with molecular *A*_m_ and solvent *A*_S_ contribution. The latter is mainly responsible for the viscosity dependence of *k*_iso_ since *A*_S_ ≫ *A*_m_ in solution. As the theoretical form *A*_S_(η) is currently unknown, we restrict ourselves by empirical results.

We have confirmed previously established power dependence *k*_iso_ ∼ η^–α^ on viscosity η in *n*-alkanes,^13^ with α = 0.30 for tS, and α = 0.35 for ttD. This can be compared to results by Fleming and co-workers,^13^ α = 0.32 for tS, and α = 0.66 for ttD. We believe that our value for ttD is correct, as the two molecules are quite similar in size and geometry that should result in similar α.

The intramolecular barrier *E*_in_ in solution is obtained from simultaneous temperature-dependent measurements of rotational and isomerization rates, eqs 7 and 8. By these equations one obtains the viscosity contribution α*E*_η_ to the apparent *E*_b_, that gives the inner barrier *E*_in_ = (*E*_b_ – α*E*_η_). We believe that this estimate is correct not only for *n*-alkanes but also for other solvents. The result for tS, *E*_in_ = 1236 cm^–1^ (Table 6) can be compared to *E*_in_ = 1398 cm^–1^ in jet. The barrier lowering by 162 cm^–1^ may be explained by the inductive/dispersive stabilization of the polar *P*-state.^69^ Previously Saltiel and Sun^16^ obtained a smaller value *E*_in_ ≈ 1000 cm^–1^ by using their solvent cage model with α = 0.4.

Despite the good power dependence τ_iso_ ∼ η^α^ in *n*-alkanes (Figure 3), large deviations occur in isoalkanes, cyclohexane and perfluorohexane (Figure 3). This indicates substantial solvent size effects which should be accounted for in theoretical consideration. We have also analyzed the isoviscosity rates^16,17^ and shown that they result in increasing barrier *E*_b_ and solvent factor *A*_S_ with decreasing viscosity η (Figure 6), that seems unphysical. However, more measurements in a wider viscosity range are required to justify this result.

## 5 Conclusions

We have discussed a longstanding problem of stilbene photochemistry, the very different photoisomerization rate *k*_iso_ of *trans*-stilbene in jet/gas and liquid solution, and the applicability of RRKM and Kramers theory to α,ω-diphenylpolienes. We have shown that in jet/gas at collisionless conditions, the RRKM rate *k*_RRKM_ = *A*_m_ exp(−*E*_in_/*kT*_m_) agrees well with experiment. A fit of *k*_RRKM_ to experimental *k*_iso_ provides key quantities, reaction frequency ν_iso_ = *A*_m_/*c* = (60 ± 5) cm^–1^ and isomerization barrier *E*_in_ = (1398 ± 26) cm^–1^. However, in compressed buffer gases and in solution, the RRKM or Kramers theory cannot fit the experimental kinetics. In this case the rate should be modified, *k*_iso_ = [*A*_m_ exp(−*E*_b_/*kT*_m_) + *A*_S _exp(−*E*_b_/*kT*_S_)], to account for the solvent collisional activation *A*_S_. A possible mechanism behind this term is that solvent collisions with tS or ttD phenyl rings provide a momentum perpendicular to the molecular plane, thus directly promoting the ethylenic twist, and hence the isomerization. Besides, measurements with high excess vibrational energy, λ_exc_ ≪ λ_00_, allow one to observe the intramolecular part *A*_m_, usually hidden in solution under the much higher *A*_S_ contribution. The *A*_m_ appears to be different from that in jet/gas and depends on solvent and deuteration pattern. The viscosity dependence of rotational and isomerization rate, *k*_R_ ∼ 1/η, *k*_iso_ ∼ η^–α^, results in the inner isomerization barrier in solution, *E*_in_ = (*E*_b_ – α*E*_η_) = (1236 ± 36) cm^–1^ for tS, where *E*_η_ is the viscosity-associated barrier.

## Supporting Information Available

Potential energy surface for stilbene photoisomerization, decay kinetics of ESA and bleach for tS in *n*-hexane and ttD in acetonitrile, geometries and vibrational frequencies for tS and ttD (PDF)
