# Supplementary material for: Photoisomerization Paths of α,ω-Diphenylpolyenes: Reaction Rate Dependence on Temperature, Excitation Wavelength, and Deuteration
Source: J Am Chem Soc. 2024 Nov 12;146(47):32463–78. doi: 10.1021/jacs.4c09134 (PMC11613511; doi:10.1021/jacs.4c09134)
Supplement: Supplementary file 1 — ja4c09134_si_001.pdf [file ja4c09134_si_001.pdf]

Photoisomerization Paths of α,ω-Diphenylpolyenes.
Reaction Rate Dependence on Temperature, Excitation Wavelength, and Deuteration

Alexander L. Dobryakov,[1] Daria Schriever,[2] Martin Quick,[3] J. Luis Pérez-Lustres,[2] Ilya N. Ioffe,[4] and Sergey A. Kovalenko[3]*

[1] N. N. Semenov Federal Research Centre of Chemical Physics, Russian Academy of Science, Moscow, 119991 Russia

[2] Department of Physics, Free University of Berlin, 14195 Germany

[3] Department of Chemistry, Humboldt University of Berlin, 12489 Germany

[4] Department of Chemistry, Lomonosov State University, Moscow, 119991 Russia

Supporting Information

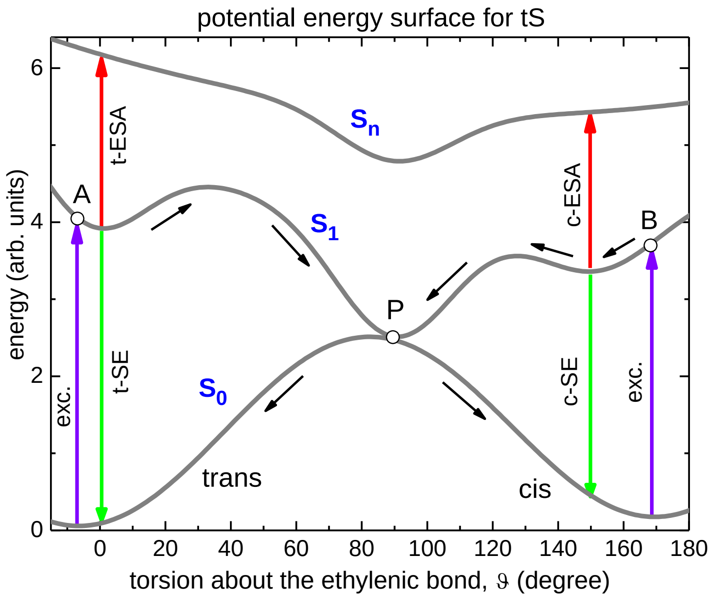

Fig. S1. Stilbene potential energy surface. When starting from the trans side, $S_0 \rightarrow S_1$ excitation creates a wavepacket that moves over a barrier to perpendicular molecular conformation P. The $S_1 \rightarrow P$ twist in n-hexane is relatively slow, τ ~100 ps, as the barrier is relatively high, E_b=1500 cm^{-1}. Subsequent relaxation $P \rightarrow S_0$, to the contrary, is ultrafast (~0.1 ps), as it is barrierless and proceeds through a conical intersection. Therefore, the trans and cis products appear in S_0 with the same time τ as the S_1 population decays. Fig. S2 confirms this for tS and ttD.

When starting from the cis side, the barrier to P is much lower, the $S_1 \rightarrow P$ twist is fast, τ~1 ps, that makes possible to observe the P-state, and watch how hot products (cis and trans) appear in the ground state S_0, and subsequently cool down by surrounding solvent [24,25].

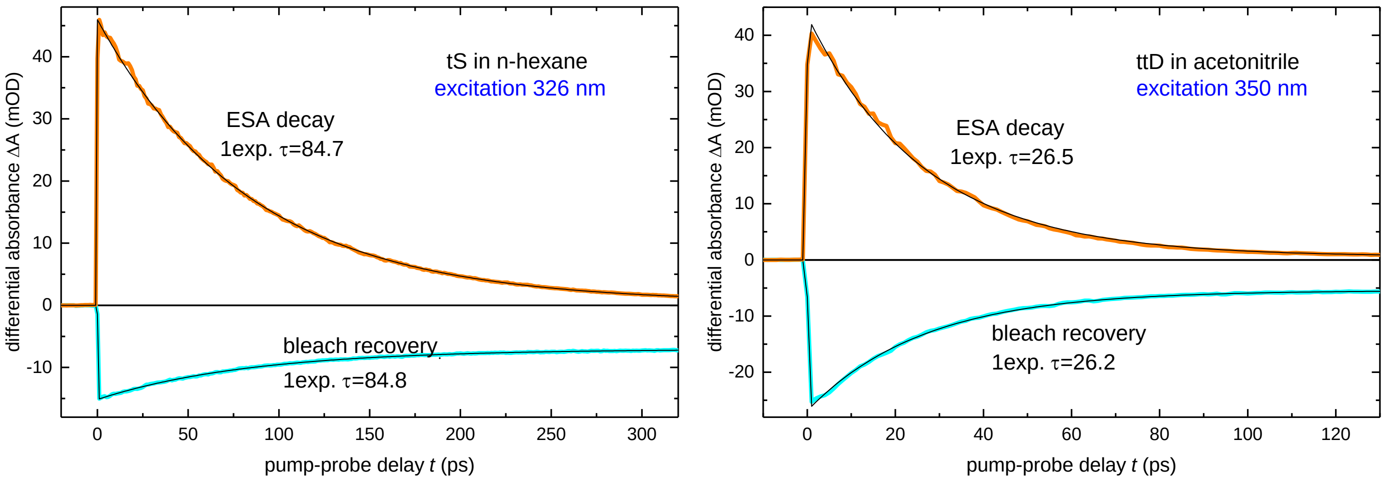

Fig. S2. ESA decay and bleach recovery kinetics upon 0-0 excitation, for tS in n-hexane, and for ttD in acetonitrile. The decay of ESA and the bleach recovery develop with the same time τ as argued in Fig. 1.

Cartesian coordinates of the ^1A_g excited state of *tt*-DPB (XMCQDPT2).

C	-1.873058923	0.336465693	0.296389495
C	1.887458761	-0.270945965	0.083697849
C	-0.592184204	-0.316106252	0.218194355
C	0.608063226	0.384696141	0.161265740
C	-3.114858857	-0.299125611	0.388896642
C	3.132967257	0.360272437	0.015943477
C	-4.326984529	0.491874814	0.462474100
C	4.342521533	-0.434882025	-0.054142318
C	-3.270060984	-1.743563694	0.412066272
C	3.293837157	1.804251425	0.011216144
C	-4.520206741	-2.313474142	0.502028006
C	4.547236526	2.370045058	-0.056347992
C	-5.691539201	-1.501827523	0.573916157
C	5.716154775	1.554476973	-0.123785213
C	-5.568160341	-0.106769682	0.552201158
C	5.587007843	0.159660496	-0.121201553
H	-1.869910108	1.422894922	0.286096383
H	1.879954156	-1.357436933	0.079194527
H	-0.551117657	-1.399077626	0.202675812
H	0.568074013	1.467735736	0.171215822
H	-4.239566614	1.572049039	0.445861939
H	4.250537710	-1.514810284	-0.052593961
H	-2.395152876	-2.376385259	0.358007372
H	2.420550359	2.439670102	0.061174769
H	-4.619463779	-3.391198013	0.518020587
H	4.650982749	3.447474308	-0.058841177
H	-6.666865868	-1.964881183	0.644095407
H	6.694071704	2.014375682	-0.176446465
H	-6.454602539	0.512652090	0.606011432
H	6.471634380	-0.462576244	-0.172358517

Cartesian coordinates of the ^1B_u excited state of *tt*-DPB (XMCQDPT2).

C	-1.864237312	0.376727415	0.319682939
C	1.844718616	-0.238440563	0.096439565
C	-0.607263215	-0.256795793	0.244196813
C	0.607160919	0.433833775	0.171845210
C	-3.116318073	-0.289490635	0.396770812
C	3.123917997	0.370160904	0.018831472
C	-4.314655345	0.469890260	0.465634494
C	4.288778294	-0.439565214	-0.049588761
C	-3.236236433	-1.720638205	0.408593201
C	3.297211976	1.795784410	0.006078111
C	-4.483868660	-2.325045421	0.484816340
C	4.568821894	2.348428462	-0.070421210
C	-5.653014916	-1.542275414	0.551240568
C	5.705150611	1.518103885	-0.136644360

C	-5.550577168	-0.140567766	0.540953015
C	5.548171565	0.119391789	-0.125307103
H	-1.878204260	1.464185540	0.318944939
H	1.818114632	-1.326353389	0.098072897
H	-0.563646874	-1.341431587	0.240634145
H	0.580819169	1.518115465	0.175239895
H	-4.243270528	1.551802599	0.458060670
H	4.173009902	-1.518008585	-0.041124294
H	-2.346552238	-2.335397019	0.359152530
H	2.431871853	2.444568827	0.055807115
H	-4.557949786	-3.405116087	0.492769334
H	4.687093690	3.424680753	-0.079372345
H	-6.624682374	-2.017571859	0.610500766
H	6.695039292	1.954563193	-0.196098219
H	-6.446101211	0.465507393	0.592057461
H	6.418734672	-0.522088904	-0.176207610

Cartesian coordinates of the transition state for photoisomerization in the S_1 (ex-^1A_g) state of *tt*-DPB (XMCQDPT2).

C	-1.532642710	0.598908511	-0.899733435
C	1.863588830	-0.251844634	0.403222197
C	-0.277903338	-0.003578508	-0.899994017
C	0.713956567	0.519754923	-0.046823951
C	-2.751804032	0.041206738	-1.417187429
C	2.882758555	0.035866848	-0.574919757
C	-3.924745274	0.834759677	-1.410160518
C	3.719860988	-0.974254289	-1.123386327
C	-2.824986475	-1.269671993	-1.958934454
C	3.072687375	1.374184531	-1.074565104
C	-4.025015312	-1.746079952	-2.476815864
C	4.033583473	1.653366589	-2.047825578
C	-5.174203638	-0.935145360	-2.468663417
C	4.853313986	0.635058329	-2.549934485
C	-5.121889729	0.341444496	-1.941434928
C	4.687631704	-0.682144434	-2.059104077
H	-1.606119358	1.580472271	-0.438216690
H	1.664132462	-1.309782610	0.577126273
H	-0.100767160	-0.970562963	-1.353857927
H	0.539574446	1.543134562	0.295262731
H	-3.883289006	1.833961470	-0.993942620
H	3.595777560	-1.992883435	-0.772248330
H	-1.949347590	-1.903893764	-1.960318996
H	2.514110772	2.190715838	-0.630935150
H	-4.074596764	-2.746170229	-2.885463658
H	4.161411237	2.671677883	-2.394288802
H	-6.101913821	-1.315919565	-2.875469750
H	5.612561273	0.851243137	-3.290639213
H	-6.006635657	0.963717684	-1.931997158
H	5.323101974	-1.475980282	-2.432970344

Cartesian coordinates of the transition state for photoisomerization in the S_2 (ex-^1B_u) state of *tt*-DPB (XMCQDPT2).

C	-1.733200466	0.508386978	-0.356693105
C	1.913652131	-0.344227690	0.175156757
C	-0.526875621	-0.175579892	-0.372317244
C	0.641814415	0.334233810	0.228433018
C	-2.970693098	0.071552767	-0.964034480
C	3.011114848	0.120168562	-0.597915165
C	-4.129770639	0.850054121	-0.786414617
C	4.256117350	-0.556053173	-0.577596129
C	-3.070846856	-1.112254156	-1.737599218
C	2.891020269	1.281897694	-1.418415553
C	-4.289650069	-1.489828659	-2.298919896
C	3.964968453	1.721959835	-2.183877159
C	-5.432882676	-0.695749760	-2.104274706
C	5.190450853	1.032062065	-2.144212985
C	-5.341631713	0.476391762	-1.346141223
C	5.320822416	-0.107303820	-1.338455962
H	-1.764547406	1.456495251	0.177293013
H	2.052361510	-1.270521742	0.740153682
H	-0.447826999	-1.135484851	-0.872734807
H	0.587143922	1.326627108	0.687810268
H	-4.064044616	1.758134000	-0.197734951
H	4.363165083	-1.437613899	0.045415007
H	-2.195193843	-1.726358033	-1.903455128
H	1.935133304	1.799407108	-1.442167475
H	-4.353737942	-2.395058620	-2.888511726
H	3.860575902	2.602085808	-2.806428730
H	-6.378728781	-0.989035411	-2.542039367
H	6.030163931	1.380735698	-2.733866458
H	-6.217724899	1.093323891	-1.194182493
H	6.262560330	-0.641238606	-1.308636970

Harmonic frequencies of D0 (tS) (XMCQDPT2)

Stationary point	Transition state
28.54 (torsional motion)	32.46 i
56.4	52.6
69.23	54.29
124.03	133.48
173.39	146.36
232.73	241.24
283.05	260.93
290.55	291.89
395.92	357.92
398.85	396.31
442.59	409.32
453.98	455.36
456.17	466.74

504.2	502.02
599.74	568.72
605.64	607.09
617.9	611.54
638.97	649.84
640.1	656.81
709.85	674.28
723.17	708
744.87	748.63
779.23	768.5
807.13	817.69
807.36	821.45
823.11	831.23
852.97	841.69
858.57	878.99
861.57	879.88
936.96	945.91
937.37	946.32
941.01	947.24
941.14	948.11
978.25	991.55
988.59	992.52
1024.56	1024.26
1025.09	1030.03
1089.81	1081.39
1095.5	1087.15
1165.7	1096.19
1165.81	1118.31
1172.1	1168.92
1183.23	1169.29
1202.24	1183.59
1225.25	1183.74
1256.46	1210.06
1317.07	1322.41
1345.85	1325.64
1384.76	1377.5
1428.3	1428.52
1431.43	1432.59
1462.26	1451.33
1506.36	1506.23
1509.87	1507.64
1519.55	1511.41
1544.74	1544.92
1548.73	1568.07
1570.57	1572.75
1616.82	1628.21
1677.82	1647.84
3146.88	3029.46
3151.34	3052.06
3184.68	3151.35
3184.77	3153.81

3193.17	3187.56
3193.19	3187.64
3204.05	3200.95
3204.9	3202.91
3214.48	3213.48
3214.8	3213.49
3224.24	3225.31
3224.65	3226.47

Harmonic frequencies of D2 (XMCQDPT2)

Stationary point	Transition state
27.97 (torsional motion)	31.74 i
56.4	52.32
68.52	54.27
123.86	132.53
171.33	145.85
226.54	223.88
257.14	228.35
282.55	283.97
394.86	348.76
396.22	392.75
419.72	404.52
434.26	428.76
443.21	447.25
492.47	462.77
575.98	518.51
598.08	604.65
604.84	610.44
610.37	614.69
628.72	641.52
648.01	663.1
668.11	664.7
730.21	744.73
747.07	750.7
785.39	779.81
785.71	793.34
799.87	817.12
801.32	819.29
856.95	878.19
857.58	878.43
936.14	890.24
936.85	896.76
937.31	945.98
940.99	946.21
942.55	947.52
975.54	948.09
978.25	992.25

991.11	993.61
1024.59	1029.29
1025.03	1029.97
1099.26	1082.39
1105.37	1087.15
1161.75	1145.73
1166.32	1169.17
1170.24	1169.82
1191.23	1182.64
1198.88	1187.03
1308.53	1315.31
1327.35	1317.94
1378.68	1370.5
1419.25	1427.73
1424.38	1431.02
1450.4	1441.38
1505.17	1504.8
1508.88	1507.22
1518.39	1509.51
1543.63	1542.28
1546.87	1567.91
1561.38	1571.73
1616.07	1627.65
1673.67	1647.08
2318.4	2223.65
2325.51	2250.34
3184.67	3150.99
3184.72	3153.57
3192.61	3187.56
3192.8	3187.64
3203.91	3200.72
3204.49	3202.9
3214.34	3213.48
3214.66	3213.49
3224.24	3225.23
3224.64	3226.47
